# Robust deepfake video detection using spatio-temporal features and dynamic difference learning

**DOI:** 10.1038/s41598-026-53545-w

**Published:** 2026-06-02

**Authors:** Eman AbdElfattah, Hamdy M. Mousa, Ashraf Elsisi, Nader Mahmoud

**Affiliations:** Computer Science Department, Faculty of Computers and Information, Minufiya University, Shebin El Kom, Egypt

**Keywords:** Deepfake detection, Spatio-temporal analysis, Feature engineering, Facial landmark detection, Engineering, Mathematics and computing

## Abstract

Recent progress in facial manipulation technologies has made deepfake videos increasingly convincing, posing significant challenges to detection systems that rely solely on analyzing individual frames. Consequently, there has been a growing emphasis on investigating spatial and temporal inconsistencies within video sequences to more accurately distinguish between genuine and manipulated content. However, many existing approaches still depend on combining frame-level and sequence-level features without adequately addressing irregularities in facial motion, which can significantly constrain detection performance. To overcome these limitations, we propose a comprehensive deep learning framework that integrates both spatial and temporal analysis. Facial landmarks are extracted from each video frame using Dlib’s 68-point detector, providing geometric descriptors of facial structure. These landmarks are fed into a Transformer encoder to capture both short- and long-term motion dynamics, enhanced by a Dynamic Difference Module (DDM) that emphasizes abrupt, unnatural changes. Meanwhile, CNNs extract spatial features, and LSTMs model temporal dependencies. The performance was assessed using standard metrics, including precision, recall, and F1-score, ensuring a comprehensive evaluation of the framework’s effectiveness. Beyond a study, the full framework was experimentally validated across three benchmark datasets—FaceForensics++ (FF++), UADFV, and DFDC—achieving a remarkable 100% accuracy, thereby demonstrating its robustness and strong generalization capability.

## Introduction

In recent times, fake videos, images, and news have spread widely, significantly affecting various fields such as journalism, politics, and digital security. False news is spread in the name of political leaders, whether in the form of written news or edited videos where faces are swapped or speakers’ voices are altered, or someone may create fake videos of another person and blackmail them financially. Leveraging advancements in deep learning, particularly generative techniques such as Variational Autoencoders (VAEs)^1^ and Generative Adversarial Networks (GANs)^2,3^ these synthetic media methods can generate highly lifelike facial forgeries by using DeepFakes, Face2Face, FaceShifter^4^ and FaceSwap^5^ techniques. This growing issue has prompted a need in developing automated detection systems aimed at spotting the subtle inconsistencies left behind by manipulation. Early deepfake detection methods primarily focused on analyzing single frames using CNNs^6,7^. These approaches achieved moderate success, but the performance has deteriorated with the rise of more advanced forgery techniques that produce increasingly realistic spatial details^8^. More importantly, these single-frame methods often failed to capture temporal relationships such as facial expressions and movement patterns which are crucial for identifying synthetic content. To address this challenge, several studies have adopted multi-frame and temporal modeling strategies^9^. Since deepfake videos are typically generated frame by frame, they often contain temporal artifacts—such as inconsistencies in facial details, expressions, and motion patterns—that even advanced generation methods fail to eliminate completely.

Other studies have focused on improving and enhancing the video preprocessing stage to better identify locations of manipulation. FastMTCNN^10^, a faster and optimized variant of the original Multi-task Cascaded Convolutional Neural Network (MTCNN), which is used for face detection and alignment with high efficiency. Dlib’s X-point landmarks^11^ are another robust and widely adopted machine learning and computer vision library that offers pre-trained models for detecting facial landmarks. These configurations include 68-point, 81-point, and 5-point landmark sets, which correspond to key facial features such as the eyes, nose, and mouth. Other tools for enhanced video preprocessing like Mediapipe Face Mesh^12^ detect 468 facial landmarks in real time, even on phones, but all that detail comes at a cost: heavier computational demands. For simpler tasks, it might be overkill, while the OpenCV’s face detectors^13^(like Haar cascades and Deep Neural Network (DNN) models) are quick and easy to set up but falter with tricky angles, bad lighting, or when fine facial details are needed. In comparison FastMTCNN and Dlib ensure efficient face detection and speed.

In this paper, we propose a deepfake detection framework that integrates FastMTCNN and Dlib for efficient facial feature extraction, along with a hybrid learning architecture that combines LSTM^14^, Transformer networks, a DDM^15^, and a Visual Geometry Group (VGG16) backbone^16^. This configuration enables the model to effectively capture both spatial and temporal inconsistencies that are indicative of manipulated facial content. To evaluate the performance of the proposed framework, we conducted experiments using three widely adopted benchmark datasets in this area: DFDC^17^, FF++, and UADFV. The results demonstrate that proposed framework achieves high accuracy, precision, recall, and F1-score, confirming its effectiveness in detecting forged videos across a range of challenging scenarios.

The main contributions of this work can be summarized as follows:

- Propose a unified deepfake detection framework that integrates spatial and temporal information in a single end-to-end pipeline.
- Considering various geometric features such as distances, angles, and ratios to capture the structural relationships between facial regions. This feature of engineering enhances the framework’s ability to detect subtle, fine-grained distortions typically introduced in deepfake videos.
- In separate experiments, both Xception and VGG16 CNN encoders were tested independently to extract high-level spatial features on a per-frame basis. Each model demonstrated strong performance and yielded high accuracy results.
- Capture short-term and long-term temporal dependencies using hybrid architecture combining LSTM and Transformer encoders.
- Employ a DDM to highlight abrupt spatial-temporal inconsistencies indicative of deepfake artifacts.

The remainder of this paper is organized as follows: Sect. "Related works" reviews the related works in deepfake detection. Section  "Proposed Framework" details the proposed framework methodology. Section "Experimental Evaluation" describes the dataset, experimental setup, and evaluation procedures. Finally, Sect. "Conclusion" presents the conclusions and outlines directions for future work.

## Related works

Facial landmarks constitute a treasure trove of geometric and motion-related information that proves invaluable for identifying deepfakes. Researchers have increasingly turned to these markers to isolate and quantify the minute temporal variations and intricate facial edits that often slip past the naked eye. Here, we summarize key contributions that exploit facial landmarks to enhance both the granularity of the analysis and the overall robustness of detection outcomes.

Qader et al.^18^ presented a hybrid approach that processes input from consecutive video frames and feeds them into the ResNet-Swish-BiLSTM model, an optimized residual network that combines convolutional layers with bidirectional LSTM (BiLSTM) for training and classification. This method is designed to effectively detect artifacts in deepfake frames that appear unnatural. Authors conducted experiments using the publicly available DFDC dataset and the FF + + dataset. This approach achieved an accuracy of 96.23% on the FF + + dataset al.one, and 78.33% when evaluated on a combined dataset from FF + + and DFDC. A key limitation of this research lies in its reliance on numerous CNN layers, which may contribute to the notably low accuracy observed when applied to the DFDC dataset combined with FF++.

Zhang et al.^19^ introduced a novel approach that improves deepfake detection by analyzing coordinated motion patterns among facial landmarks. It introduces a coordinated motion landmarks mining strategy and a landmark temporal dynamic relation module (LTDRM) to effectively capture these patterns and detect signs of forgery. Incorporating LTDRM into different video-based detection models enhances their generalization ability, achieving significant Area Under Curve (AUC) improvements on the Celeb-DF and DFDC datasets. This method achieved an accuracy of 90.70% on the Celeb-DF dataset, and 74.33% when evaluated on DFDC. According to the results obtained by the authors, their model is not capable of absolute generalization to a larger number of videos with more diverse features.

Tian et al.^20^ proposed a new way to find deepfakes that used the MixStyle technique and several loss functions at the same time. Mixstyle is used for improving frameworks able to endure by integrating incorporating information (such as mean and variance) between the the sample used for training. This method uses Cross-Entropy Loss, ArcFace Loss, and Focal Loss to make the model better at telling the difference between things. This lets it better capture complex forging patterns and deal with data distributions that aren’t balanced. Furthermore, the use of MixStyle introduces variability in visual styles during training, which promotes stronger generalization across diverse datasets and manipulation types. This model is trained on four datasets FaceForensics++, CDF2, DFD, and DFDC, where it uses three critical metrics area under curve (AUC), equal error rate (EER), and average precision (AP) to measure performance. They achieved AUC scores of 99.68%, 94.85%, 99.12%, and 80.81% across the four datasets, respectively. A closer look at the results reveals that the model performed the worst on the DFDC dataset. This suggests that the model is effective primarily when working with high-quality video content.

Haliassos et al.^21^ introduced an approach called LipForensics for detecting forged face videos. It concentrates on inconsistencies in semantically complex mouth movements by leveraging detailed representations obtained through lip-reading. LipForensics achieved leading generalization performance on previously unseen forgery types and exhibits superior robustness against common corruption compared to other techniques. They achieved AUC scores of 82.4% and 73.5% when training on the Celeb-DF-v2 and DFDC datasets, respectively. However, a notable limitation of the approach is its focus solely on lip movements, without considering other important facial features.

Wodajo et al.^22^ described an enhanced Deepfake video detector based on a Convolutional Vision Transformer (CViT2), expanding on the ideas from their previous work (CViT). CViT architecture combines two key parts a CNN that learns and extracts useful features from videos, and a Vision Transformer that analyzes and classifies those features through an attention mechanism. They trained and tested the model on five different datasets DFDC, FF + + Celeb-DF v2, DeepfakeTIMIT, and TrustedMedia. On test data that the model didn’t see during training, it achieved accuracy 95% on DFDC, 94.8% on FF++, 98.3% on Celeb-DF v2, and 76.7% on TIMIT. The model works well with Celeb-DF v2 dataset as the resolution of videos in this dataset are 480p and have 30 frames in second, this provides enough detail to capture subtle changes over time. However, when applied to a dataset that contains only audio like DeepfakeTIMIT, the model’s performance significantly declined. Moreover, the lower the video quality, the less effective the model became.

Solaiman et al.^23^ presented a deep learning model based on a CNN designed specifically to classify frames from deepfake videos. This model delivers strong performance, as shown through detailed testing on the VDFD dataset, where it achieved excellent results with an average precision of 95%, recall of 94%, and an F1-score of 94%. Beyond the VDFD dataset, the model also demonstrates its effectiveness across several well-known deepfake datasets, including FF++, Celeb-DF, and DFDC. On these datasets, it maintains solid average precision, recall, and F1-scores, ranging from 80% to 85% for frame-level detection. The research results are too limited for the model to achieve good generalization.

Malik et al.^24^ proposed a new method for detecting deepfake videos. The research draws on the DFDC and FaceForensics datasets. As part of preprocessing, they performed frequency-based frame extraction on each video to better capture important details by using OpenCV library. For the detection itself, they combined CNNs with LSTM networks in a hybrid CNN-LSTM approach. This method achieved 82% accurate results based on the FF + + dataset in distinguishing fake videos from real ones and 72% accurate results based on DFDC dataset. The research findings indicate that the method demonstrates low efficiency and remains ineffective in accurately detecting forgeries.

Mad Sahar et al.^25^ employed the advanced image processing algorithm InceptionResNetV2 to detect deepfakes in video content. InceptionResNetV2 combines the strengths of both ResNet and Inception architetures, which give it amazing accuracy. It looks likely to become one of the strong candidates for deepfake detection in future. The team developed an Android App which mashes all this. They used MIT App Inventor to do the build, and Google Colab for the prediction model. To assess the app’s effectiveness, they tested it using three different models, InceptionResNetV2, EfficientNetB, and ResNet50 on two widely used datasets, DFDC and CelebDF, comprising a total of 400 video samples. This model achieves 93.20% accuracy on the DFDC dataset and an impressive 97.72% on CelebDF. In contrast, ResNet50 performed the worst on DFDC, with an accuracy of just 52.28%, while EfficientNetB0 had the lowest score on CelebDF, at 30.81%. The test model accuracy is 71.19%, this makes the model unable to detect fake videos outside its training data, in addition, it lacks the ability to extract spatio-temporal details from the images, focusing solely on face detection and cropping.

Sheng et al.^26^ proposed RetinaFace detector, EfficientNet-B and Multi-Attention Mechanism that breaks down a target object into several local parts, analyzes each part separately, and then pieces together their relative positions to identify the object’s category. This strategy helps reduce reliance on identity specific information and allows the model to better capture subtle texture details. To further boost performance, they also incorporate a forgery trace detection module that pinpoints manipulated regions and blends global and texture features. This ensures the model pays closer attention to local details without depending heavily on identity cues. Extensive experiments and thorough analyses on the FaceForensics++, Celeb-DF, and DFDC datasets show that this method delivers AUC results 99.9% ,72.04, 95.5% in sequence. This model may face challenges when processing images with very high compression or have busy and complex backgrounds.

Xiong et al.^27^ presented a deepfake detection framework called the BiLSTM Multi-Head Self-Attention Network (BMNet). This approach combines a Bi-Directional Long Short-Term Memory Network (BiLSTM), which captures the temporal relationships between video frames, with a Multi-Head Self-Attention Mechanism (MHSA) that focuses on features from different regions of each frame. This allows BMNet to better identify both dynamic and localized forgery clues. In the experiments, they extract features from 68 facial landmarks in every video frame and test the method across four datasets UADFV, FF++, Celeb-DF, and DFDC. BMNet achieves impressive accuracies of 95.54%, 92.18%, 80.20%, and 84.72% on the FF++, UADFV, Celeb-DF, and DFDC datasets, respectively, according to the previous results, the model is unable to detect forgeries when tested on new videos that were not part of the dataset it was trained on.

Yin et al.^28^ proposed a new dynamic difference learning approach designed to tell apart the subtle differences between changes caused by face manipulation and those resulting from natural facial movements. They developed two key components, the Dynamic Fine grained Difference Capture module (DFDC-module) and the Multi scale Spatio-Temporal Aggregation module (MSA-module). The DFDC-module uses a self-attention mechanism along with fine-grained denoising techniques to filter out variations due to normal facial movements, producing long-range difference attention maps that focus on potential forgeries. Meanwhile, the MSA-module gathers temporal information from multiple directions and scales, helping the model understand complex spatio-temporal patterns. This approach was trained on the FF++, Celeb-DF, and DFDC datasets, achieving accuracies of 98.9%, 99.6%, and 94.14%, respectively. While it excels at capturing temporal information between frames, it falls short in fully leveraging the spatial details within each individual frame.

Hernandez-Ortega et al.^29^ introduced DeepFakesON-Phys fake video detection method, that is built around a Convolutional Attention Network (CAN) to capture both spatial and temporal cues from video frames. By analyzing and integrating both types of information, the model becomes more effective at identifying deepfakes. To evaluate its performance, the authors tested DeepFakesON-Phys on two of the most recent and widely used public datasets in the field: Celeb-DF and DFDC Preview. The model achieved 98.7%, 94.4% accuracy on both datasets in sequence. This fake detection method can’t deal with types of face manipulations it hasn’t encountered during training, also it doesn’t integrate the temporal information across video frames which are used to detect other forms of facial manipulations, like face morphing.

Zhou et al.^30^ proposed CEPrompt, a cross-modal framework for facial expression recognition that integrates visual and textual modalities using vision-language pretrained models such as CLIP. It introduces the Emotion Conception-guided Visual Adapter (EVA) to capture category-specific visual features guided by textual emotion concepts and the Conception-Appearance Tuner (CAT) to enhance interaction between emotion concept prompts and appearance prompts, improving multimodal feature alignment. Knowledge distillation is used to preserve the generalization ability of the pretrained model during fine-tuning. Experiments on multiple real-world FER datasets demonstrate that CEPrompt achieves state-of-the-art performance, highlighting the benefit of incorporating semantic emotion cues into visual representations. However, the method relies on a pretrained CLIP backbone and prompt tuning, which increases computational complexity, its effectiveness outside standard FER benchmarks—such as extreme poses, occlusions, or cross-domain scenarios—has not been fully explored, and some design choices, including prompt structures and tuning strategies, may require careful validation in different conditions.

Zhou et al.^31^ propose UA-FER, an uncertainty-aware representation learning framework for facial expression recognition (FER) that integrates vision–language pre-training (VLP) models (e.g., CLIP) with Evidential Deep Learning (EDL) to improve accuracy and robustness in challenging real-world scenarios. The method introduces Multi-granularity Feature Decoupling (MFD) to decouple global and local emotion-related representations using image–text affinity and a Relation Uncertainty Calibration (RUC) module to model and correct uncertain visual–textual relationships, thereby reducing overconfident mispredictions and enhancing emotion-discriminative features. Extensive experiments on in-the-wild and in-the-lab FER benchmarks show that UA-FER surpasses state-of-the-art models, demonstrating its effectiveness in handling ambiguous cases and complex variations. However, the approach depends on pretrained VLP models, which increases computational overhead, and may require careful tuning of uncertainty calibration to generalize well across diverse datasets and real-world conditions.

Zhou et al.^32^ IncepTR is a hybrid framework proposed for micro-expression recognition (MER) that integrates Inception-CBAM and Vision Transformer architectures to capture both local and global facial features. The model uses an Inception network enhanced with CBAM attention to extract multi-scale spatial features, while a Vision Transformer (ViT) captures long-range dependencies and global contextual information. The extracted features from both branches are fused to improve the representation of subtle facial movements. Experimental evaluations on benchmark datasets such as CASME II, SAMM, and SMIC demonstrated competitive performance compared with existing MER approaches. The main advantage of this method is the combination of CNN-based local feature extraction with transformer-based global modeling, although the approach increases model complexity and still suffers from the limited size of micro-expression datasets.

Zhou et al.^33^ proposed a Dual-Branch Attention Network (Dual-ATME) for micro-expression recognition (MER) that combines handcrafted knowledge with deep attention mechanisms to improve feature representation. The model consists of two components: Hand-crafted Attention Region Selection (HARS), which extracts features from manually selected facial regions based on prior knowledge, and Automated Attention Region Selection (AARS), which automatically learns informative regions using an attention mechanism. The features from both branches are then fused using a similarity-based strategy for final classification. Experiments on benchmark datasets such as CASME II, SAMM, SMIC, and MEGC2019-CD demonstrated competitive performance compared with state-of-the-art MER methods. The main advantage of this approach is the integration of handcrafted and deep features to enhance discriminative representation. However, the method still relies partially on manually selected regions and limited datasets, which may affect its generalization ability.

Wu et al.^34^ proposed the Adaptive Patch Contrast (APC) framework, which significantly advances ViT-based Weakly Supervised Semantic Segmentation (WSSS) by using Adaptive-K Pooling (AKP) and Patch Contrastive Learning (PCL) to enhance patch embeddings, improve intra-class consistency, and separate inter-class features. Its single-stage, end-to-end design increases training efficiency and generates higher-quality pseudo-labels. Experiments on PASCAL VOC 2012 and MS COCO 2014 demonstrate that APC outperforms prior state-of-the-art methods. However, the approach is limited to standard benchmark datasets, relies on computationally intensive ViT architectures, and its generalization to more diverse or real-world scenarios has not yet been validated.

Wu et al.^35^ proposed TEMA-LLM, a cross-domain sequential recommendation framework that integrates Large Language Models (LLMs) with a tag-enriched multi-attention mechanism. The model utilizes LLMs to generate semantic tags from item content, which are combined with multimodal features, including textual, visual, and ID representations, to enhance item embeddings. Furthermore, the proposed multi-attention strategy effectively captures both intra-domain and inter-domain user preferences, leading to improved modeling of user behavior across domains. Experimental results demonstrate that the method outperforms several state-of-the-art approaches, indicating its effectiveness in handling data sparsity and improving recommendation accuracy. Despite these advantages, the model depends on LLM-generated tags that may introduce noise or bias, and its complex architecture may increase computational cost and limit scalability in real-world dynamic settings.

Wu et al.^36^ introduced a weakly supervised semantic segmentation framework based on contrastive prompt clustering, aiming to improve pixel-level prediction with limited annotations. The method leverages prompt-based learning to generate semantic cues, which are further refined using a contrastive clustering strategy to enhance feature discrimination and spatial consistency. By effectively utilizing weak supervision signals, the proposed approach reduces reliance on dense pixel annotations while maintaining competitive segmentation performance. Experimental evaluations demonstrate that the model achieves improved accuracy compared to existing weakly supervised methods, highlighting its ability to capture meaningful semantic structures. The main strengths of this work include its efficient use of limited supervision, enhanced feature representation through contrastive learning, and improved segmentation consistency. However, the method may suffer from sensitivity to prompt design, potential instability in clustering quality, and increased computational overhead due to the contrastive learning process, which could limit scalability in large-scale or real-time applications.

Wu et al.^37^ proposed an image augmentation agent for weakly supervised learning, aiming to enhance model performance by automatically selecting effective augmentation strategies. The approach employs a learning-based agent that dynamically generates and applies augmentation policies to improve feature generalization under limited supervision. By integrating augmentation optimization into the training process, the method reduces dependency on manual design and enhances robustness to data variability. Experimental results demonstrate that the proposed framework improves performance compared to conventional augmentation techniques, particularly in scenarios with scarce labeled data. The main advantages of this work include its adaptive augmentation strategy, improved generalization capability, and reduced need for manual intervention. However, the method introduces additional training complexity, requires careful tuning of the augmentation agent, and may incur higher computational cost, which could limit its applicability in large-scale or real-time systems.

**Fig. 1:**
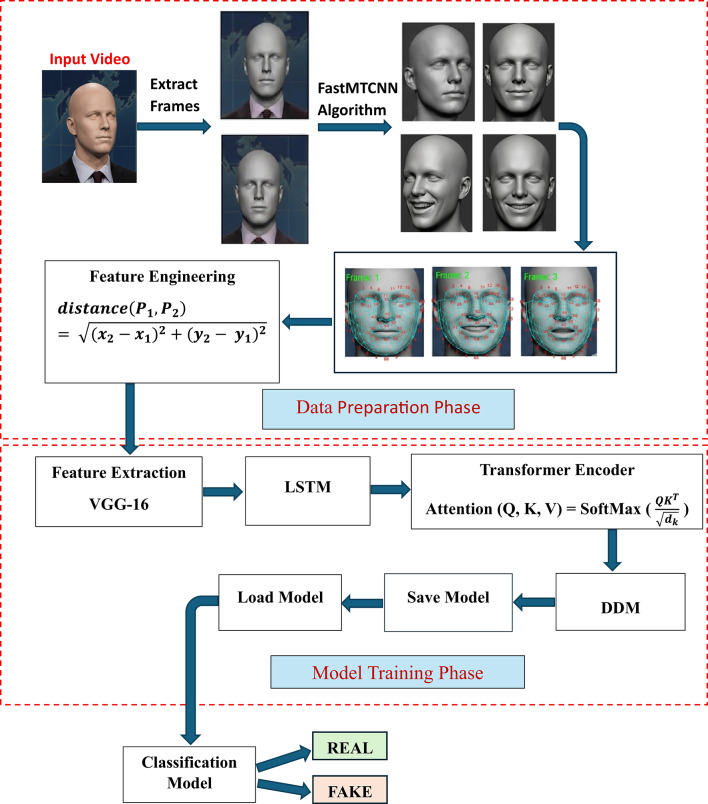
Overview of the proposed deepfake detection framework architecture.

## Proposed framework

This paper introduces a comprehensive deepfake detection framework comprising three main stages: the data preparation phase, the model training phase, and the classification phase. Each stage is carefully designed to perform specific tasks that are essential for accurately detecting manipulated video content. In the data preparation phase, input videos are converted into frames, where facial regions are detected using FastMTCNN, frames are enhanced, facial landmarks are annotated, and geometric features are computed. In the training phase, these features are processed through a pipeline consisting of LSTM, a Transformer encoder, and a DDM, followed by model saving. Finally, during the classification phase, the trained model is loaded and used to classify new input videos as either real or fake. The overall workflow of the proposed framework is illustrated in Figure 1.

### Data preparation phase

#### Frame extraction

Upon receiving the input video, the first step involves decomposing the continuous video stream into a sequence of discrete image frames. This conversion enables frame-level analysis and facilitates downstream processing such as face detection and feature extraction. Each extracted frame is treated as an independent image sample and passed to the next stage in the pipeline. The entire set of video frames is used to guarantee temporal clarity and detail. These frames are then fed into a fast and efficient face detection algorithm FastMTCNN which identifies and localizes facial regions with high accuracy and speed. This early step ensures that only relevant facial areas are processed in subsequent stages, thereby reducing computational overhead and enhancing model focus on tamper-prone regions.

#### Face detection and alignment

**Fig. 2:**
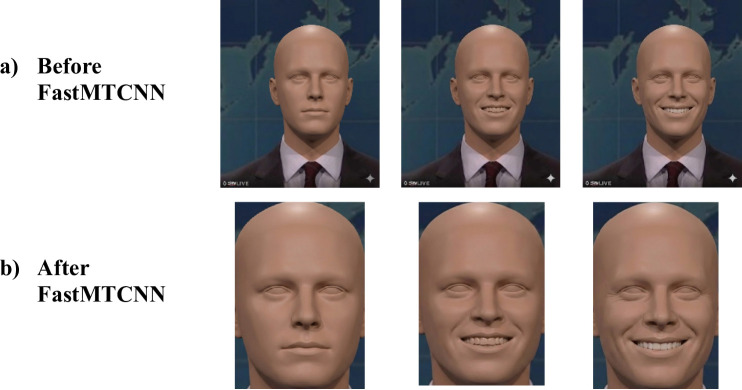
Example video frames: (**a**) before applying FastMTCNN, and (**b**) after face detection and processing using FastMTCNN.

FastMTCNN^37^ performs face detection using a streamlined three-stage pipeline. It all starts with preprocessing, resizing images at multiple scales to handle faces of varying sizes. In the first stage, the Proposal Network (P-Net) quickly scans the image to identify potential facial regions. These initial candidates are subsequently forwarded to the Refine Network (R-Net), which filters out false positives and further adjusts the bounding boxes to enhance detection accuracy. The final refinement is handled by the Output Network (O-Net), which not only confirms the face regions but also pinpoints key facial landmarks such as the eyes, nose, and corners of the mouth. To further improve precision, Non-Maximum Suppression (NMS) is applied to remove redundant or overlapping boxes. FastMTCNN improves upon the original MTCNN by incorporating GPU acceleration and batch processing, significantly speeding up detection without compromising accuracy. These enhancements make it well-suited for real-time applications. Examples of frame outputs are presented in Figure 2, where Figure 2 (a) shows frames before face detection and Fig. 2 (b) displays the results after applying FastMTCNN.

#### Frame enhancement

To improve the quality of the detected facial regions, several enhancement techniques are applied. First, the image is converted from the RGB color space to YCrCb^38^, which is a color format that breaks down an image into its brightness (Y) and color components (Chroma-red (Cr) and Chroma-blue (Cb), according to Eq. 1:

1 $$\:\left[\begin{array}{c}Y\\\:Cr\\\:Cb\end{array}\right]=\:{f}_{color}(R,\:G,\:B)$$

Where Y is the brightness channel, Cr measures how much the red component in the image stands out compared to the overall Y. Cb measures how much the blue component in the image stands out compared to the overall Y.

This technique separates the brightness channel (Y) from the color components, making it easier to adjust contrast more effectively. Then, Contrast Limited Adaptive Histogram Equalization (CLAHE)^39^ is applied to enhance local contrast in small regions of the image, while keeping noise amplification under control, as shown Eq. 2:

2 $$\:h\left(i\right)=\:\sum\:_{(x,y){\upvarepsilon\:}tile}\delta\:(I\left(x,y\right)-i)$$

Where $\:\delta\:$(.) is the Kronecker delta:

3 $$\delta (k)\, = \,\left\{ {\begin{array}{*{20}c} {1,} & {if\,k = 0} \\ {0,} & {otherwise} \\ \end{array} } \right.$$

The image I(x, y), with a resolution of M×N pixels, is divided into smaller sections known as tiles or contextual regions. After adjusting the Y, it is merged back with the original Cr and Cb (color) channels and converted back to the RGB color space, as demonstrated by the Eq. 4:

4 $$\:RGB=\:{f}_{color}^{-1}\:(\acute{Y}\:,Cr,\:Cb)$$

**Fig. 3:**
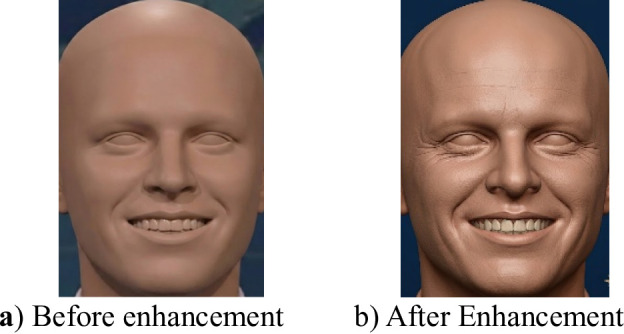
Illustration of frame enhancement: (**a**) the original frame prior to processing, and (**b**) the enhanced frame, where lighting conditions and edge details are significantly improved.

A Non-Local Means Denoising Filter (NLM)^40^ is an advanced image processing technique designed to reduce noise while preserving fine details and textures. Unlike traditional filters that rely only on nearby pixels, NLM enhances image quality by comparing and averaging similar patches from across the entire image, regardless of their spatial distance. Finally, to enrich the visual appearance, the image is converted to the Hue Saturation Value (HSV)^41^ color space, where both saturation (S) and brightness (V) levels are increased before converting the image back to RGB format. This last step boosts color vibrancy and overall visibility, resulting in a more vivid and perceptually enhanced facial image, as shown in Figure 3.

#### Facial landmark annotation

**Fig. 4:**
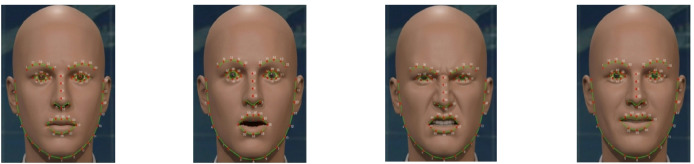
Example of facial landmark annotation using dlib’s 68-point model.

After the face is extracted and enhanced through the aforementioned preprocessing steps, we employ a shape predictor model^42^ from Dlib library to estimate the precise (x, y) coordinates of 68 specific facial landmarks as depicted in Eq. 5, for covering regions like the jawline, eyebrows, eyes, nose, and lips shown in Figure 4.

5 $$\:{P}_{i\:}=\left({x}_{i},\:{y}_{i}\right),\:\:for\:i=\mathrm{0,1},\dots\:.,67\:$$

where a$\:\:{x}_{i}$ and $\:{y}_{i}\:$represent the horizontal and vertical pixel positions of the $\:{\mathrm{i}}^{\mathrm{t}\mathrm{h}}$ landmark point. Each landmark index corresponds to a standardized facial region: points 0–16 map the jawline, points 17–21 correspond to the right eyebrow, points 22–26 to the left eyebrow, points 27–30 to the upper part of the nose, points 30–35 to the lower part of the nose, points 36–41 represent the right eye, points 42–47 the left eye, points 48–59 the outer part of the mouth, and points 60–67 the inner part of the mouth. Thus, the landmark detection process relies on a learned model rather than explicit geometric formulas, enabling robust and automatic identification of facial features in images or video frames.

#### Feature engineering on landmarks

In the feature extraction stage of the proposed framework, we calculate geometric features like distances and angles between facial landmarks. This helps capture detailed spatial relationships and maintain the structural consistency of the face, which can be vital for spotting subtle signs of manipulation. Distances and angles play a vital role in feature engineering for facial landmarks because they help capture the meaningful geometry of a face in a way that’s stable and reliable. Considering only the raw coordinates of landmarks is not enough, since those numbers can easily change if the face moves, turns, or appears larger or smaller. Instead, measuring distances like how far apart the eyes are, or the gap between the nose and mouth gives us a sense of the face’s true proportions, no matter its size in the image. Likewise, the angles formed between key facial points—like those between the eyes and nose or the corners of the mouth—help convey the overall shape, orientation, and even expressions of the face. These angles can reveal subtle asymmetries or unnatural changes that might suggest tampering, like in deepfake videos. Altogether, these geometric features act like a unique fingerprint for a face, giving machine learning models a much stronger foundation for telling real from fake or for recognizing different facial attributes. Distances are calculated using the Euclidean distance between points: $\:{P}_{1}=\left({x}_{1},{y}_{1}\right)\:,\:\:{P}_{2}=({x}_{2},\:{y}_{2})$^26^ as given by Eq. 6:

6 $$\:distance\left({P}_{1},\:{P}_{2}\right)=\:\sqrt{{({x}_{2}-{x}_{1})}^{2}+{({y}_{2}-\:{y}_{1})}^{2\:\:\:}}$$

This equation is designed to capture key facial geometry, including measurements such as the distance between the eyes, the spacing between the nose and mouth, the width of each eye, the width and height of the mouth, the vertical length of the face from nose to chin, and the horizontal width from cheek to cheek. Angles are computed to capture the orientation and geometric layout of facial components using the angle between two vectors. Given three points A, B, and C, where B is the vertex of the angle, the angle θ is calculated through Eq. 7^43^:

7 $$\:\theta \: = arccos(\frac{{\vec{B}A\:.\:\vec{B}C\:\:}}{{\left\| {\vec{B}A} \right\| \cdot \left\| {\vec{B}C} \right\|}}\:\:$$

Here, $\:\overrightarrow{B}A$ and $\:\overrightarrow{B}C$ are vectors formed from point B to A and from B to C, respectively. This is used to compute angles such as between the left eye, nose tip, and right eye.

### Model training phase

**Fig. 5:**
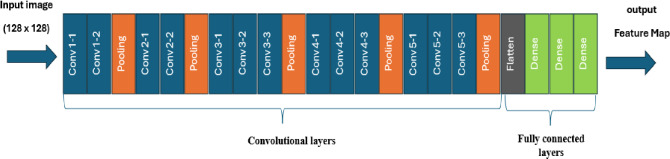
Illustration of the VGG16 architecture, comprising stacked convolutional and pooling layers followed by fully connected layers.

This stage begins with feature extraction, to extract deep hierarchical features such as edges, textures, shapes, and object parts from video frames. The VGG16 model has been utilized, as it is a widely adopted deep convolutional neural network known for its simple and uniform architecture, consisting of 16 layers with small 3 × 3 convolutional filters, as shown in Figure 5. In this work, the fully connected classification layers are removed. Our framework accepts input images of size 128 × 128. The output is stored in feature maps, which play a crucial role in allowing the model to interpret and represent image content through a hierarchical structure.

We chose VGG16 as the primary backbone for our experiments for several reasons. First, its relatively simple and uniform architecture makes it easy to integrate with additional modules such as LSTM, Transformer Encoder, and the Dynamic Difference Module, without adding excessive complexity. Second, despite being older than more recent architectures, VGG16 provides robust feature extraction capabilities, particularly for facial landmark-based spatial features. Finally, our empirical experiments confirmed that VGG16 consistently outperformed alternative backbones like ResNet50 and EfficientNet on our datasets, achieving the best overall accuracy. These factors motivated our decision to use VGG16 in the proposed framework. When the output of VGG16 is passed to an LSTM network, the goal is to analyze the temporal dynamics across a sequence of video frames. While VGG16 extracts rich spatial features from each individual frame—such as edges, shapes, and object structures, as shown by Eq. 8

8 $$\:F = \{ f_{1} ,f_{2} , \ldots \:,f_{T} \} \:,\,f_{t} \:\varepsilon \:R^{d}$$

**Fig. 6:**
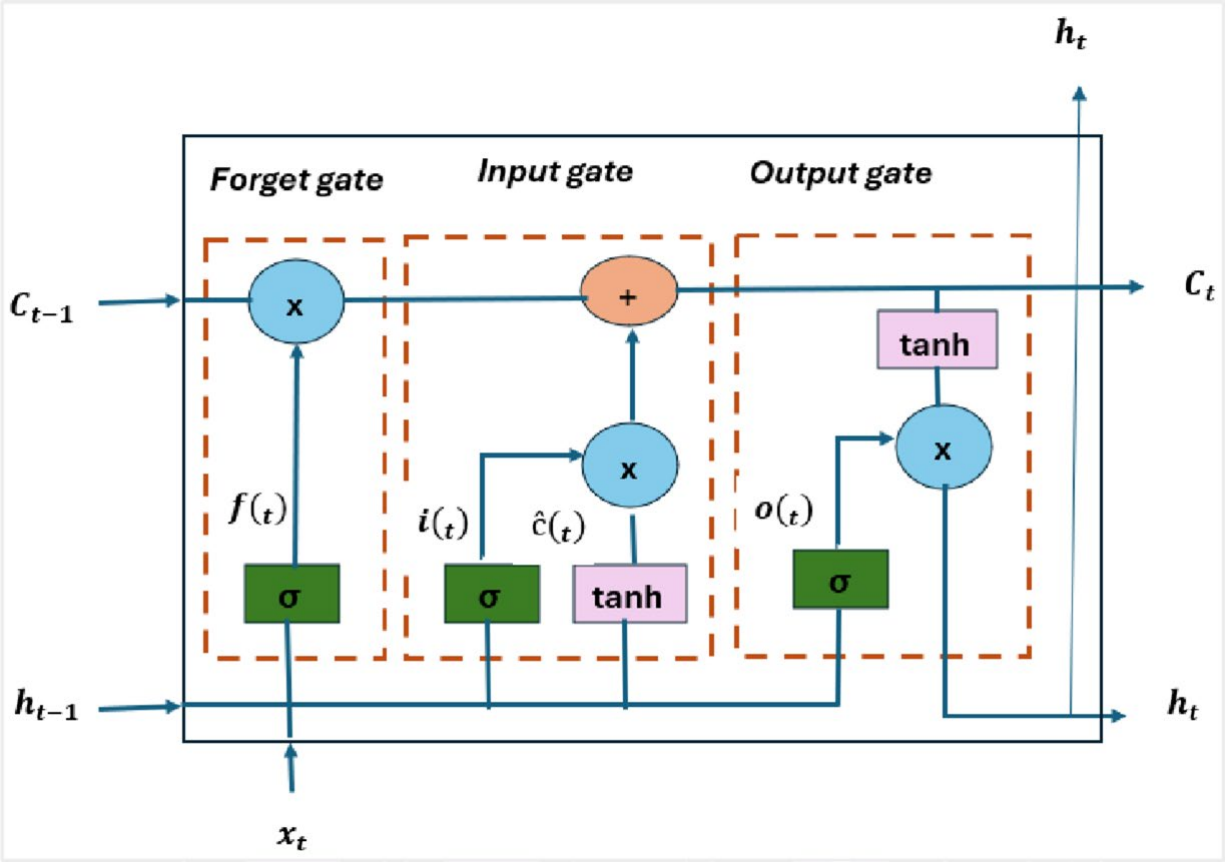
Illustration of the LSTM architecture^14^.

The feature vector was extracted from the $\:t$-th frame of the video. Here, $\:T$is the total number of frames, and $\:d\:$represents the dimensionality of the feature vector, capturing the visual information of each frame. This representation allows the model to process temporal dynamics by analyzing the evolution of features across consecutive frames. LSTM focuses on understanding how these features change over time as shown in Figure 6. It processes the sequence of frame-level feature vectors and learns patterns, trends, or abrupt shifts that may occur throughout the video. Where subtle inconsistencies between frames can indicate manipulation. Depending on its configuration, the LSTM can output a sequence of hidden states ($\:{h}_{t}$) which capture information at each time step $\:t$, the LSTM takes the frame-level feature $\:{f}_{t}$and the hidden state from the previous step $\:{h}_{t-1}$as inputs, producing the updated hidden state $\:{h}_{t}$,as given by Eq. 9.

9 $$\:{h}_{t}=LSTM\:({f}_{t},{h}_{t-1})$$

Here, $\:h_{t} \:\varepsilon R^{k}$ represents the hidden state at frame $\:t$, encoding both the current frame information and the temporal context from preceding frames. Collectively, the sequence of hidden states across all frames is denoted as $\:{H}_{LSTM}=\{{h}_{1},{h}_{2},\dots\:.,{h}_{T}\}$ which captures the local temporal evolution of the video, allowing subsequent modules to leverage these dynamics for tasks such as classification or anomaly detection. After obtaining the sequence of hidden states from the LSTM, $\:{H}_{\mathrm{LSTM}}$, we enhance the representation by adding positional encoding (PE) to retain information about the order of frames. This gives the input to the Transformer encoder as Eq. 10.

10 $$\:X\:=\:{H}_{LSTM}+PE$$

**Fig. 7:**
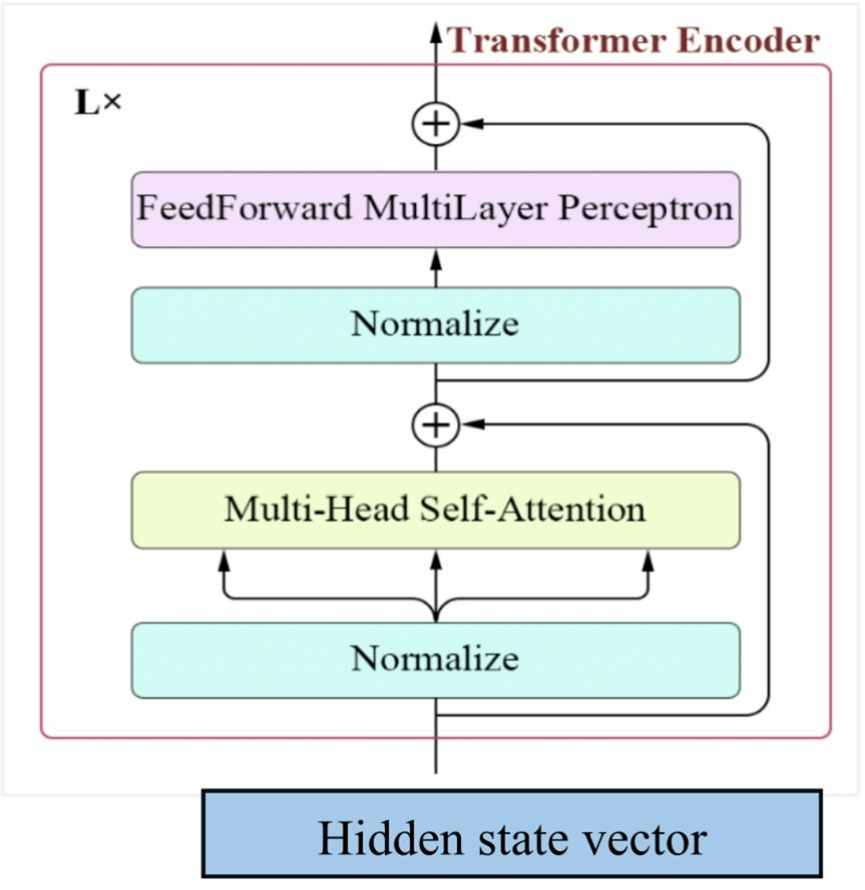
Illustration of the Transformer encoder architecture.

As shown in Figure 7, the transformer then applies the Multi-Head Self-Attention mechanism to capture long-range dependencies between frames. First, the input $\:\boldsymbol{X}$ is projected into queries $\:Q$, keys $\:K$, and values $\:V\:\:$using learnable weight matrices. $\:Q=X{W}_{Q}\:,\:\:K=X{W}_{K}\:\:,$ $\:V\:=X{W}_{V}.$ The attention output is computed as shown by Eq. 11.

11 $$\:Attention\:\left(Q,K,V\right)=Softmax\:\left(\frac{Q{K}^{T}}{\sqrt{{d}_{k}}}\right)\:V$$

By applying multiple attention heads and concatenating their outputs, we obtain the final multi-head attention representation by Eq. 12.

12 $$\:Z = Multi\,Head\left( {Q,K,V} \right) = \left\{ {\:z_{1} ,\:z_{2} , \ldots \: \ldots \:,z_{T} } \right\},\:z_{t} \:\varepsilon \:R^{k}$$

Each $\:{z}_{t}\:$encodes not only the local temporal context captured by the LSTM but also the global relationships among all frames, providing a rich, temporally aware feature for downstream tasks. The frame-wise differences of the Transformer outputs. Specifically, for each time step $\:t=2,\dots\:,T$, the difference vector $\:{d}_{t}\:$is defined as Eq. 13.

13 $$\:{\:d}_{t}=\mid\:{z}_{t}-{z}_{t-1}\mid\:$$

where $\:{z}_{t}\:\:$and $\:{z}_{t-1}\:$are the Transformer-encoded features of the current and previous frames, respectively. Collectively, the sequence of these difference vectors is denoted as: $\:D=\{{d}_{2},{d}_{3},\dots\:,{d}_{T}\}$. This representation emphasizes sudden changes or inconsistencies across frames, which are particularly useful for detecting anomalies or manipulations in videos, such as deepfake artifacts. temporal differences. For each time step $\:t=2,\dots\:,T$, we concatenate the Transformer-encoded feature $\:{z}_{t}$with the corresponding frame difference $\:{d}_{t}$to obtain the enhanced representation by Eq. 14.

14 $$\:{\widehat{z}}_{t}=\mathrm{Concat}\left({z}_{t},{d}_{t}\right)$$

The complete sequence of these integrated features is then expressed as: $\:\widehat{Z}=\left\{{\widehat{z}}_{2},\dots\:,{\widehat{z}}_{T}\right\}.$By merging the global context captured by the Transformer with the local dynamic inconsistencies highlighted by the difference vectors, this representation provides a comprehensive and temporally aware feature set that is well-suited for tasks such as deepfake detection or other video analysis applications.

Algorithm1 illustrates and summarizes the architecture of transformer encoder, start with input numbers of attention heads (num_heads) and dimension of the feed forward layer (key_dim=ff_dim) then create multi head attention layer which allows the model to focus on different parts of the input sequence simultaneously. This layer is built using the specified num_heads, and the key. Dimension is set to ff_dim. The algorithm constructs several essential components used in the Transformer Encoder, including Feed-Forward Network (FFN) [44], which is a dense network that processes the output of the attention mechanism to introduce non-linearity and learn complex patterns. Dropout Layer is used to prevent overfitting by randomly disabling some neurons during training. Normalization Layer helps stabilize and speed up training by standardizing the inputs. Dense Layers fully connected layers that further process the data. Among the key operations in this process is the Self-Attention mechanism, mathematically represented by Eq. 15:

15 $$\:Attention\:\left(Q,K,V\right)=softmax\:\left(\frac{Q{K}^{T}}{\sqrt{{d}_{k}}}\right)V$$

where *Q*(Query), *K*(Key) and *V*(Value) matrices, which are created by transforming features extracted from video frames. For each frame or time step in the video sequence, the model generates a query that tries to find relevant information from other frames in the same video and compared to all the keys using a dot product $\:Q{K}^{T}$ to measure how similar each frame is to others. These similarity scores are scaled by dividing by the square root of the dimension of the keys $\:\sqrt{{d}_{k}}$ to keep the values stable and avoid extremely large numbers during training. The SoftMax function then converts these scores into probabilities that sum to one, indicating how much attention the model should pay to each frame in relation to the current one. Finally, these attention weights are used to compute a weighted sum of the values (*V*), effectively allowing the model to gather and focus on the most relevant information across different frames. This self-attention mechanism helps the Transformer Encoder understand temporal relationships and subtle changes across video frames. Such understanding is crucial for detecting inconsistencies or manipulations.

**Algorithm 1:**
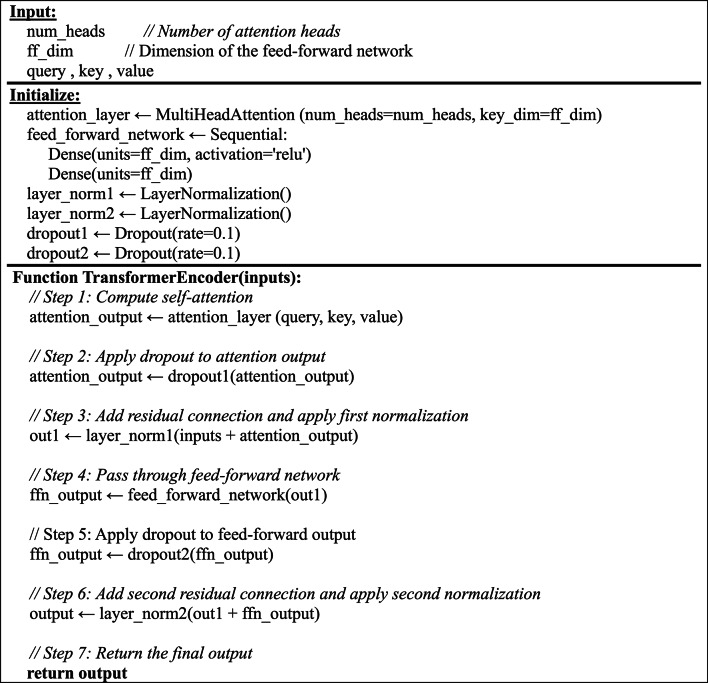
Transformer Encoder pseudocode

To reduce the overfitting the output of self-attention is passed to a feed-forward network, which is used to independently apply the same non-linear function to every position in the input sequence, shown by Eq. 16:

16 $${\mathrm{FFN}}\left( {\mathrm{p}} \right) = W_{2} \left( {ReLU\left( {W_{1} p + b_{1} } \right)} \right) + b_{2}$$

Where *p* is the input vector, W is the weight matrix, $\:{W}_{1}$ (weight matrix for first dense layer), $\:{W}_{2}$ (weight matrix for second dense layer), $\:{b}_{1}$ the bias vector for the first layer, $\:{b}_{2}$ the bias vector for the second layer. The output of FFN is passed through another dropout then followed by a second residual connection and layer normalization. Finally, the processed output is returned as the final output of the encoder block, ready to be passed to the next layer DDM. The output of the transformer encoder is processed by the DDM, a specialized component designed to highlight and focus on frame-level variations and dynamic inconsistencies that are characteristic of forged content. This module enhances the model’s sensitivity to unnatural transitions and sudden changes in facial geometry; Eq. 17 shows the process of DDM.

17 $$\:Z=\:\frac{1}{T-1}\:\sum\:_{t=1}^{T-1}\left|{S}_{t+1}-\:{S}_{t}\right|$$

Where S € $\:{\mathbb{R}\mathbb{\:}}^{T.D},$ X is the input sequence, T is time steps, D is the features. Where $\:{S}_{t}$ € $\:{\mathbb{R}\mathbb{\:}}^{D}$, $\:{S}_{t}$ is the feature vector at time steps. The result (*Z*) summarizes the average temporal change per feature across the entire sequence.

When the output of the Transformer Encoder is passed to the DDM, the goal is to detect subtle and abrupt temporal changes across consecutive frames. The Transformer Encoder provides context-aware feature representations for each frame, capturing both local and global temporal dependencies. DDM takes these enriched features and focuses specifically on the differences between frames, identifying unusual or inconsistent transitions that may indicate manipulation. This module emphasizes dynamic variations—such as unnatural movements or inconsistencies in facial expressions—that are often signs of deepfake content. The result is a refined set of features that highlight critical temporal discrepancies, which are then fed into a final classification layer to determine whether the video is real or fake. Finally, the insights the model learns are saved to create the trained model, wrapping up the training process.

**Fig. 8:**
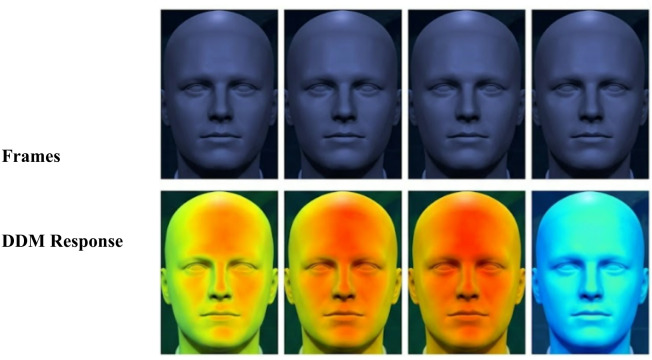
DDM attention maps highlighting motion inconsistencies across frames.

Figure 8 illustrates the sequence of frames and its attention map, The false frame shows different areas with color (yellow and red), especially around the mouth, nose, and eyes. These regions are marked as suspicious, indicating the existence of unusual artifacts that are frequently added during the deepfake production process. This visualization confirms that the DDM effectively captures subtle anomalies across frames, contributing to accurate deepfake detection. In the case of the real frame, the DDM attention map predominantly displays colors (blue and green), indicating the absence of suspicious regions and high consistency in facial features. At the end of the model training phase, the model is saved so that it can be used in the next stage, which is the classification phase. This allows us to make predictions on any fake videos that the model has not encountered during training.

### Classification phase

In the classification phase, the trained model is employed to evaluate new, unseen video data and determine its authenticity. The input video undergoes the same preprocessing pipeline as in the training stage, including frame extraction, face detection, landmark annotation, and feature engineering. These processed features are then passed through the trained sequence model comprising LSTM, Transformer, and the Dynamic Difference Module then save the model. To generate a prediction the saved model has loaded, outputs a confidence score or probability indicating whether the input video is real or manipulated. To convert these probabilities into discrete class labels, a threshold value of 0.5 is applied. This means any prediction above 0.5 is classified as class 1 (positive), while anything below or equal to 0.5 is considered class 0 (negative). This thresholding technique is a standard approach in binary classification tasks. After converting probabilities into binary labels, a classification report is printed, the final decision is made, and the video is classified as either real or fake. This phase plays a critical role in deploying the framework in real-world scenarios, enabling reliable and automated detection of deepfake content.

## Experimental evaluation

In this section we present the experimental evaluation conducted to assess the performance of the proposed framework. The experiments are designed to validate the effectiveness of the approach on benchmark deepfake datasets using various performance metrics. We detail the experimental setup, including the datasets used, ablation experimental, and evaluation criteria. The results are analyzed to demonstrate the model’s capability in accurately distinguishing between real and fake videos, and to compare its performance with existing state-of-the-art methods. Our proposed framework is trained on Google Colaboratory, a cloud-based environment that works smoothly with Jupyter Notebooks. Commonly referred to as Colab, it serves as a central tool for machine learning education and research. It provides a runtime designed specifically for deep learning, including GPU and TPU. A TPU (Tensor Processing Unit) is a custom-built processor developed by Google specifically for accelerating machine learning tasks, especially those using TensorFlow. TPUs are designed to perform large-scale matrix operations quickly and efficiently, making them ideal for training and running deep learning models. They are typically accessed through Google Cloud and offer higher speed and energy efficiency for certain AI workloads compared to GPUs, we use TPU access to boost computational performance.

### Dataset description

**Fig. 9:**
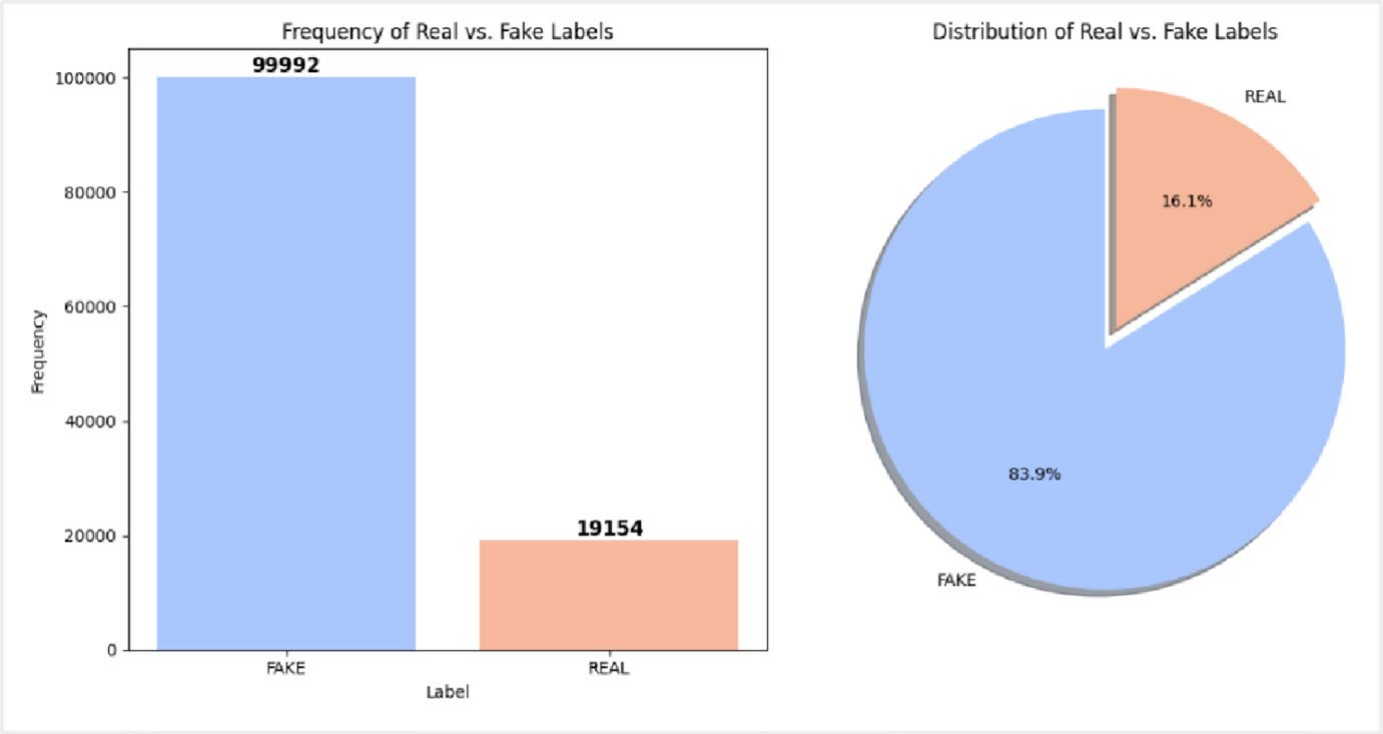
Distribution of real and fake videos in the DFDC, highlighting the class imbalance present in dataset.

We conducted our experiments using three datasets: DFDC, Face Forensics ++, UADFV. The DFDC dataset, publicly available on Kaggle, consists of 119,146 video 19,154 authentic and 99,992 manipulated, created with the participation of 3,426 volunteers, as illustrated in Figure 9.

We selected the DFDC dataset due to the complex challenges it poses for deepfake detection. The videos vary significantly in quality, with diverse facial expressions, lighting conditions, and background settings, all of which complicate the process of feature extraction. Moreover, manipulations are highly realistic, often indistinguishable from authentic videos, even to the human eye. The dataset also presents a class imbalance, with a disproportionate number of real versus fake videos, which can potentially bias the training process.

The FF++ dataset is one of the most widely used benchmarks for deepfake detection research. It contains over 1,000 original high-quality videos that have been manipulated using several popular face manipulation methods, including DeepFakes, Face2Face, FaceSwap, and NeuralTextures. In total, the dataset provides more than 5,000 manipulated videos, with a combined size exceeding 470 GB when stored in full resolution. One of the main challenges of FF++ lies in its diversity of manipulations and compression levels: the videos are available in raw (uncompressed), lightly compressed (HQ), and strongly compressed (LQ) versions. This makes detection more difficult, since real-world fake videos are often distributed on social media in compressed formats. Furthermore, the dataset includes variations in lighting, pose, background, and facial expressions, which increase the complexity of the task and provide a realistic benchmark for evaluating the robustness of deepfake detection models.

The UADFV dataset (University at Albany DeepFake Video) is among the first publicly available resources for deepfake detection. It contains a total of 98 videos, evenly divided into 49 real and 49 fake clips, with an average duration of approximately 11–15 s per video and an overall size of about 4 GB. Although relatively small compared to large-scale benchmarks such as FF++ or DFDC, UADFV introduces important challenges for detection systems. The videos exhibit variability in terms of resolution, compression artifacts, lighting conditions, and head poses, which reflect real-world recording environments. Furthermore, the limited number of samples makes it prone to overfitting and constrains its ability to represent the diversity of manipulation techniques, thus positioning it as a challenging yet valuable benchmark for evaluating the generalization capability of deepfake detection models.

The proposed framework was designed to address many of these challenges. To mitigate issues related to video quality and lighting variability, we applied frame enhancement techniques during the preprocessing stage. To address the dataset imbalance, we randomly selected an equal number of real and fake samples to create a balanced training set. This research enhanced the framework’s ability to generalize effectively across both classes. We split the dataset to 80:20 ratio, with 80% of the videos used for training and the remaining 20% held out for testing to evaluate the model’s performance.

### Evaluation metrics

In our experiments, to evaluate the performance of the proposed model architecture, we employ standard evaluation metrics, including Precision, Recall, F1-Score, and Accuracy.

These metrics provide a comprehensive assessment of the model’s classification capabilities. The definitions of these metrics are given in Eqs. (18–21):

Accuracy: This is the most often used and straightforward categorization metric. It is calculated as the number of correct predictions divided by the number of samples. Although high accuracy is typically desirable, in some cases where the class distribution is not symmetric, it may not be an informative evaluation. In these scenarios, precision, recall, and F1 score offer a more thorough assessment of model performance

18 $$\:Accuracy\:\left(ACC\right)=\:\frac{TP+TN}{Total\:EquationNumber\:of\:predictions}\:\:$$

Precision: The proportion of correctly identified deepfake videos (True Positives, TP) to the total number of videos predicted as deepfakes by the model (TP + FP). This metric highlights how often the model raises false alarms. A lower number of false positives indicates higher precision and more reliable detection.

19 $$\:\:\:\:\:Precision\:\left(P\right)=\frac{TP}{TP+FP\:}$$

Recall: The sensitivity of the model, representing its ability to correctly detect deepfake videos. It is the ratio of correctly predicted deepfakes (TP) to the total number of actual deepfakes in the dataset (TP + FN). Higher recall means fewer deepfakes are missed.

20 $$\:\mathrm{R}\mathrm{e}\mathrm{c}\mathrm{a}\mathrm{l}\mathrm{l}\:\left(\mathrm{R}\right)=\frac{\:TP}{TP+FN}$$

F1-Score: The F1 Score represents the harmonic mean of precision and recall, offering a balanced measure of the model’s performance in scenarios where both false positives and false negatives carry significant impact. Within the context of deepfake detection, this metric is particularly valuable since both the erroneous acceptance of fake content and the misclassification of genuine videos can have critical consequences.

21 $$\:\:\:\:\:\:\:\:\:\:F1\:=\:2\:x\left(\frac{\left(PxR\right)}{\left(P+R\right)}\:\right)\:$$

### Computational complexity and runtime performance

To evaluate the practical applicability of the proposed framework, we analyze its computational complexity and runtime performance in terms of training cost, inference efficiency, and overall model behavior. Our proposed framework was trained using various combinations of batch sizes (4, 8, 16, 32, 64) and epochs (10, 12, 16, 30) with an initial learning rate of 0.001. Dropout of 0.5 was applied after each fully connected layer to reduce overfitting, and data augmentation techniques such as random rotation, flipping, and brightness adjustment were used to improve generalization. Early stopping based on validation loss was employed to prevent overfitting. For the DFDC dataset, the best results were obtained using 12 epochs and a batch size of 32. For FF++, the optimal performance was achieved with 30 epochs and a batch size of 64. Similarly, for UADFV, the highest accuracy was reached using 30 epochs and a batch size of 64. The average training time per epoch was approximately 35 min, resulting in a total training time of around 7 h. Although the proposed framework integrates multiple deep learning components, including a CNN backbone, LSTM layers, and a Dynamic Difference Module (DDM), the training process remains feasible within a reasonable time budget for research and development purposes. In terms of performance, the model shows stable convergence behavior. The training accuracy improves significantly from 48.9% in the first epoch to nearly 100% in later epochs. The best validation accuracy achieved is 98.7%, with precision and recall both reaching 100% on the evaluated dataset, suggesting strong performance under the experimental conditions in detecting deepfake videos.

Regarding inference efficiency, the model processes video samples in batches, which enhances computational efficiency and reduces prediction time per sample. This batch-based processing strategy allows the framework to handle multiple video inputs simultaneously, making it potentially suitable for batch-based processing scenarios, although further validation in real-world deployment settings is required. To further reduce computational overhead, a fixed number of frames (60 frames) is sampled from each video instead of processing the entire sequence. This design choice significantly decreases the required computation while preserving the temporal information necessary for accurate classification. Overall, despite combining multiple modules, the proposed framework achieves a favorable trade-off between detection performance and computational cost within the experimental setup, indicating potential for practical applications pending further optimization.

### Quantitative evaluation

To rigorously evaluate the effectiveness of the proposed deepfake detection framework, we benchmarked its performance against several state-of-the-art methods reported in recent studies. These works differ in terms of preprocessing strategies, network architectures, and datasets, offering a broad spectrum of comparative insights. As summarized in Table 1, the results reveal substantial variability in detection performance across benchmark datasets. For example, the approach in^22^, which integrates MTCNN with CVIT2, demonstrated consistently strong results, achieving 95% on DFDC, 94.84% on FF++, and 98.25% on Celeb-DF. Conversely, the CNN (19-layer) with LSTM model proposed in^23^ underperformed, yielding only 81% on DFDC, 80.28% on FF++, and 75% on Celeb-DF. Similarly, the hybrid LSTM+CNN framework in^24^ produced weaker results, particularly on DFDC (72%) and FF++ (82%). In contrast, the InceptionResNetV2-based model in^25^ emerged as a competitive baseline, reaching 93% accuracy on DFDC and 97.7% on Celeb-DF. These findings underscore that while some architectures generalize well across datasets, many suffer from limited adaptability and may not fully capture diverse forgery patterns across datasets.

More advanced architecture has demonstrated further performance gains. For instance, the study in^26^, which utilized EfficientNet-B with a multi-attention mechanism, achieved 98.6% accuracy on DFDC and 98.8% on FF++, though its effectiveness declined markedly on Celeb-DF (72% AUC). The BiLSTM with multi-head self-attention proposed in^27^, leveraging 68-point Dlib landmarks, attained 84.7% on DFDC, 95.54% on FF++, 80.20% on Celeb-DF, and 84.72% on UADFV. Similarly^28^, combined Xception with DFDC and MSA modules, producing 94% accuracy on DFDC, 98.9% on FF++, and an impressive 99.7% on Celeb-DF. In another study^29^, integrated 81 Dlib-specific landmarks with a Convolutional Attention Network (CAN), obtaining 94% on DFDC and 98.7% on Celeb-DF.

**Table 1:** Comparison between the proposed framework and state-of-the-art methods.

Study	Preprocessing method	Techniques	Dataset			
			DFDC	FF++	Celeb-DF	UADFV
			ACC	ACC	ACC	ACC
Wodajo et al.^22^	MTCNN	CVIT2	95%	94.84%	98.25%	–
Solaiman et al.^23^	ImageData Generator	CNN 19 layer **+** LSTM	81%	80.28	75%	–
Malik et al.^24^	opencv	LSTM **+** CNN	72%	82%	–	–
Mad Sahar et al.^25^	ComputerVision DataLibrary	InceptionResNetV2	93%	–	97.7%	–
Sheng et al.^26^	RetinaFace detector	EfficientNet-B **+** Multi-Attention Mechanism	98.6%	98.8%	72% (**AUC**)	–
Xiong et al.^27^	Dlib’s 68 point landmarks	BiLSTM **+** Multi-Head Self-Attention Network	84.7%	95.54%	80.20%	84.72%
Yin et al.^28^	MTCNN	Xception **+** DFDC Module **+** MSA Module	94%	98.9%	99.7%	–
Hernandez-Ortega et al.^29^	Dlib’s 81 specific landmarks	Convolutional Attention Network (CAN)	94%	–	98.7%	–
Proposed framework	FastMTCNN+Dlip’s 68 landmarks	LSTM **+** VGG16 **+**Transformer Encoder +DDM	**100%**	**100%**	–	**99.6%**

In contrast, our proposed framework—FastMTCNN with 68-point Dlib landmarks, LSTM, VGG16, and a Dynamic Difference Module (DDM)— achieved higher performance on the evaluated datasets compared to the selected baselines. Notably, it reached 100% accuracy on DFDC and FF++ under the evaluated experimental conditions, along with 99.6% accuracy on UADFV, indicating competitive performance across the evaluated datasets. Furthermore, as illustrated in Table 1, previous studies reported lower performance across these datasets. For instance, on DFDC, the highest reported accuracy among prior works was 98.6% by Sheng et al., while our framework achieved 100%. On FF++, the best previous result was 98.9%, compared to 100% accuracy achieved under our experimental conditions. Moreover, on UADFV, prior works reported a maximum accuracy of 84.72%, whereas the proposed approach improved the performance to 99.6%. These results suggest the effectiveness of our framework in enhancing deepfake detection performance across multiple benchmark datasets.

**Fig. 10:**
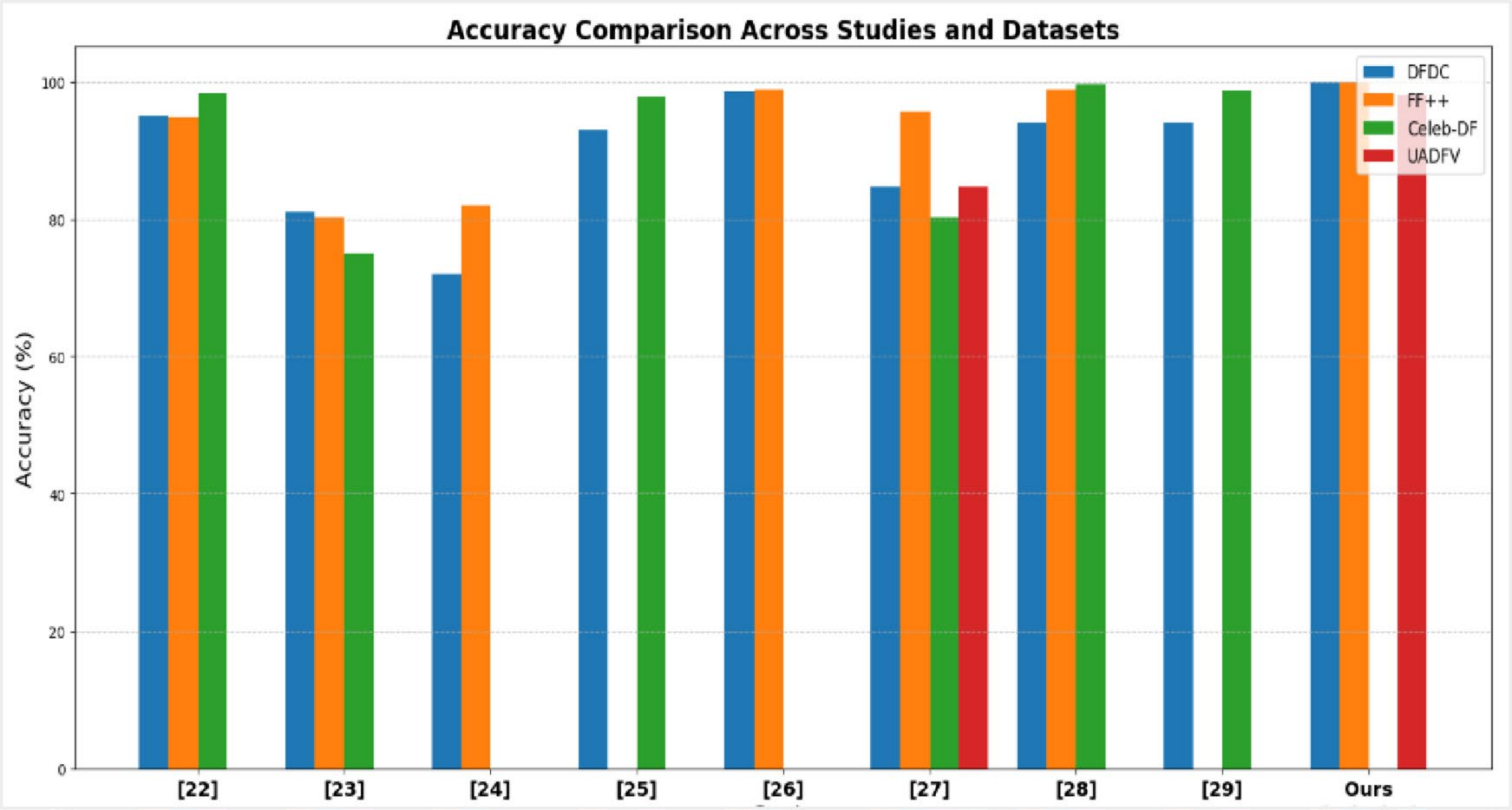
Accuracy comparison across DFDC, FF++, Celeb-DF, and UADFV datasets for various state-of-the-art methods, showing higher accuracy compared to the reported methods and suggesting promising generalization within the evaluated datasets.

The results presented in Figure 10 highlight distinct performance disparities among existing deepfake detection methods across the widely used datasets—DFDC, FF++, Celeb-DF, and UADFV. Simpler architectures, such as CNN+LSTM^23^ and LSTM+CNN^24^, struggled to deliver reliable performance, with accuracy levels remaining comparatively low. More advanced models, including EfficientNet-B with attention^26^ and Xception combined with MSA^28^, demonstrated stronger results but continued to exhibit noticeable variability depending on the dataset, indicating limitations in generalization. These results indicate stable performance across the evaluated datasets, indicating competitive performance compared to previously reported approaches.

### Comparative study of network architectures

In this section, we present the results of experiments conducted using randomly selected video samples to compare two backbone networks: VGG16 and Xception. The evaluation process began with testing the FastMTCNN algorithm in isolation, and then progressively integrating additional components, including Dlib’s 68-point facial landmarks, the transformer encoder, and the Dynamic Difference Module (DDM). Both VGG16 and Xception were assessed under these configurations to measure their effectiveness. Across all experimental setups, VGG16 outperformed Xception across the evaluated configurations, indicating improved feature representation and detection accuracy. The experiments were performed on three benchmark datasets: DFDC, FF++, and UADFV—with the comprehensive results systematically reported in Table 2, 3

### Results on DFDC dataset

Table 2 summarizes the experimental results obtained using two CNN architectures, VGG16 and Xception, these results obtained using FastMTCNN for face detection and evaluated on varying sizes of DFDC dataset. Each experiment was conducted with over 30 training epochs. The datasets comprised both real and fake videos, organized into two scales: a smaller set of 10,000 videos (5,000 real and 5,000 fake) and a larger set of approximately 39,000 videos (19,154 real and 20,000 fake). On the smaller dataset, VGG16 achieved an accuracy of 84%, which improved to 90% on the larger dataset. Similarly, Xception reached 85% accuracy on the smaller set and 89% on the larger one. These results indicate that increasing the dataset size generally leads to better accuracy.

**Table 2:** Experimental results using FastMTCNN only and DFDC dataset.

Dataset scale	CNN-network	
	VGG-16	Xception
10 K [ 5000 reals, 5000 Fakes]	84%	85%
~39 K [19154 reals, 20000 Fakes]	90%	89%

**Table 3:** Experimental results on three datasets: DFDC, FF++, UADFV Using FastMTCNN Combined with Dlib’s 68 Landmarks, Transformer Encoder, and DDM, evaluation in terms of Accuracy (%), Precision (%), Recall (%), F1- Score (%), Bold and underlined values indicate the best performance

CNN-netwoark	DataSet	Epochs	Videos	Accuracy	Precision	Recall	F1
Xception	DFDC	10	60	59%	60%	86%	71%
			200	**93%**	95%	90%	92%
		12	400	89%	93%	85%	89%
VGG16		10	60	99%	98%	99%	98.5%
			200	100%	100%	100%	100%
		12	400	**100%**	100%	100%	100%
VGG16	FF++	16	200	98%	99%	98%	98.5%
		30	400	**100%**	100%	100%	100%
VGG16	UADFV	30	98	**99.6%**	99.5%	99.7%	97%

Table 3 presents the experimental results of the deepfake video detection framework integrating FastMTCNN for face detection, Dlib’s 68 facial landmarks for geometric feature extraction, a Transformer Encoder for temporal modeling, and a DDM module to highlight abrupt changes. The table compares various configurations based on the CNN backbone (Xception vs. VGG16), number of training epochs between 10 and 30, and dataset size. The batch size was dynamically adjusted between 4 and 64 depending on the number of videos in each dataset. Increasing the batch size leads to more stable and potentially faster training steps, while increasing the number of epochs allows the model to learn more thoroughly from the data. When both are adjusted together in a balanced way, they enhance the training of deeper or more complex models more effectively. The comparison is consistently conducted across the three previously mentioned datasets: DFDC, FF++, and UADFV.

**Fig. 11:**
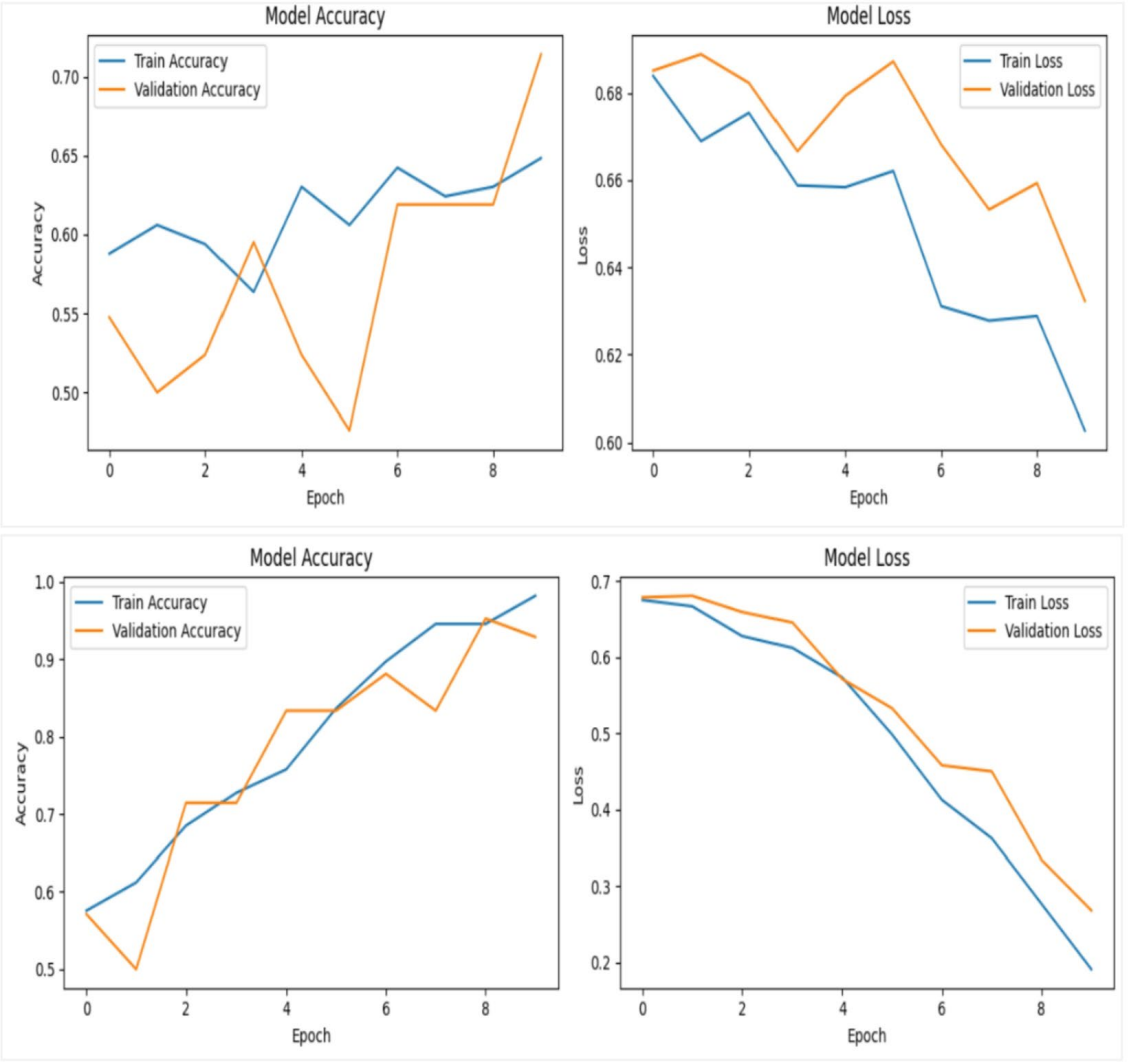
Training and validation performance of the deepfake detection framework using Xception (top row) and VGG16 (bottom row) on the DFDC dataset. Each model was trained for 10 epochs on 60 videos with a batch size of 4; the right-hand plots show steadily decreasing training and validation loss across epochs.

In the first experiment, we evaluated the performance of two backbone networks, Xception and VGG16, using the same sample of 60 videos evenly divided between 30 real and 30 fake. Both models were trained for 10 epochs with a batch size of 4. The Xception network achieved a training accuracy of 59%, while its validation accuracy fluctuated noticeably before stabilizing around 70%, as shown in the top row in Figure 11. In contrast, the VGG16 network reached a significantly higher training accuracy of 99%, with validation accuracy also exhibiting fluctuations but ultimately stabilizing near 93%, as illustrated in second row in Figure 11. For both models, the loss curves (displayed on the right side of the figures) show a steady decrease in training and validation loss over time.

**Fig. 12:**
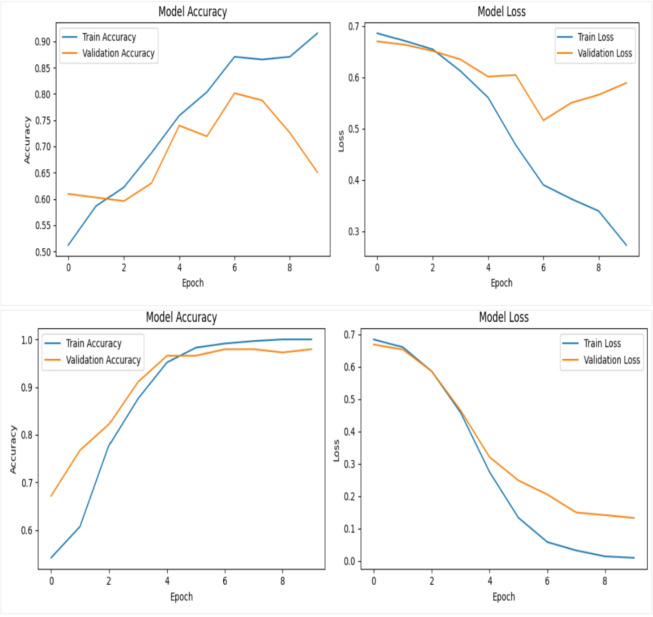
Illustrates the training and validation performance of the deepfake detection framework using Xception (first row) and VGG16 (second row) on the DFDC dataset. Each model was trained for 10 epochs using 200 videos with a batch size of 8. In both cases, the corresponding loss curves show a consistent and steady decrease for both training and validation sets across epochs, indicating stable convergence and effective learning behavior.

In the second experiment, we have tested both the Xception and VGG16 networks on a slightly larger scale of dataset videos, evenly split between 100 real and 100 fake. Both models were trained for 10 epochs with a batch size of 8. The Xception network achieved a training accuracy of 93%, but its validation accuracy fluctuated considerably before stabilizing around 65%, as shown in first row in Figure 12. Conversely, the VGG16 network reached a training accuracy of 100%, with validation accuracy also showing fluctuations but ultimately settled near 98%, as illustrated in second row of Figure 12. For both models, the loss curves (displayed on the right side of the figures) demonstrate a steady decline in training and validation loss over time.

**Fig. 13:**
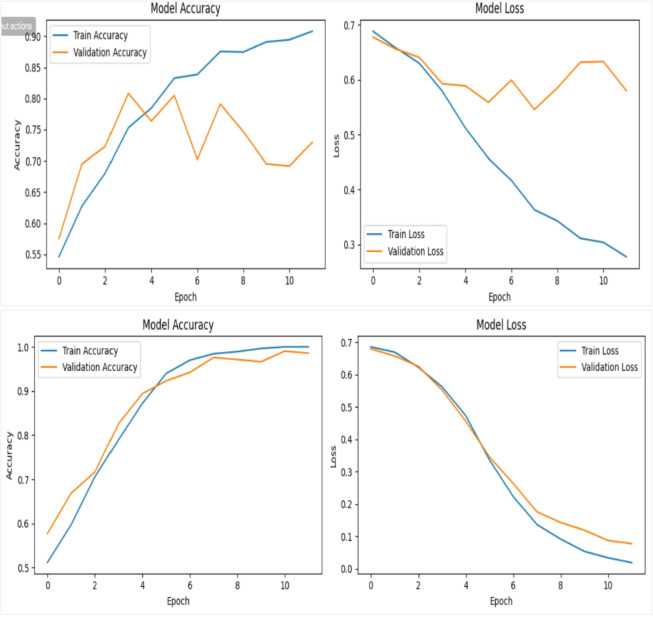
Illustrates the training and validation performance of deepfake detection models using Xception (first row) and VGG16 (second row), each trained for 12 epochs on 400 videos with a batch size of 32; in both cases, the right-side graphs display steadily decreasing training and validation loss curves across epochs.

In the final experiment, both the Xception and VGG16 networks were evaluated using a dataset of 400 videos, evenly split between 200 real and 200 fake. The models were trained for 12 epochs with a batch size of 32. The Xception network achieved a training accuracy of 89%, while its validation accuracy fluctuated significantly before stabilizing around 73%, as illustrated in top row in Figure 13. In contrast, the VGG16 network reached a perfect training accuracy of 100%, with validation accuracy showing some variation but ultimately settling near 99%, as shown in the second row of Figure 13. For both models, the loss curves (displayed on the right side of the figures) indicate a consistent decrease in training and validation loss over time.

The overall findings indicate that increasing the dataset size and training epochs leads to improved accuracy and stability. More importantly, across DFDC dataset and experimental conditions, VGG16 consistently outperformed Xception, indicating that VGG16 is more suitable than Xception within the tested configurations for facial feature extraction in our proposed framework.

### Validation of proposed framework on FF++ dataset

**Fig. 14:**
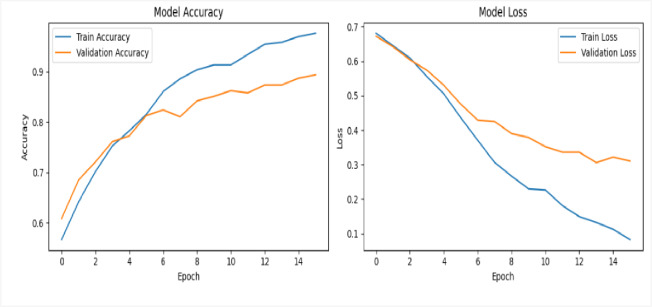
Illustrates the training and validation performance of deepfake detection framework using VGG16, trained for 16 epochs on 200 videos with a batch size of 64 on FF++ dataset; the right-side graphs display steadily decreasing training and validation loss curves across epochs.

We validated our proposed deepfake detection framework’s efficacy and generalizability by conducting additional evaluation on a benchmark dataset, specifically FF++. This cross-dataset validation is useful for assessing the model’s robustness and its capacity to perform accurately on data beyond its initial training distribution. In the first experiment, using VGG16 network was evaluated using a dataset of 200 videos, evenly split between 100 real and 100 fake. The framework was trained for 16 epochs with a batch size of 64. As illustrated in Figure 14, the VGG16 network reached a perfect training accuracy of 98%, with validation accuracy showing some variation but ultimately settled near 89%. The loss curves (displayed on the right side of the figures) indicate a consistent decrease in training and validation loss over time.

**Fig. 15:**
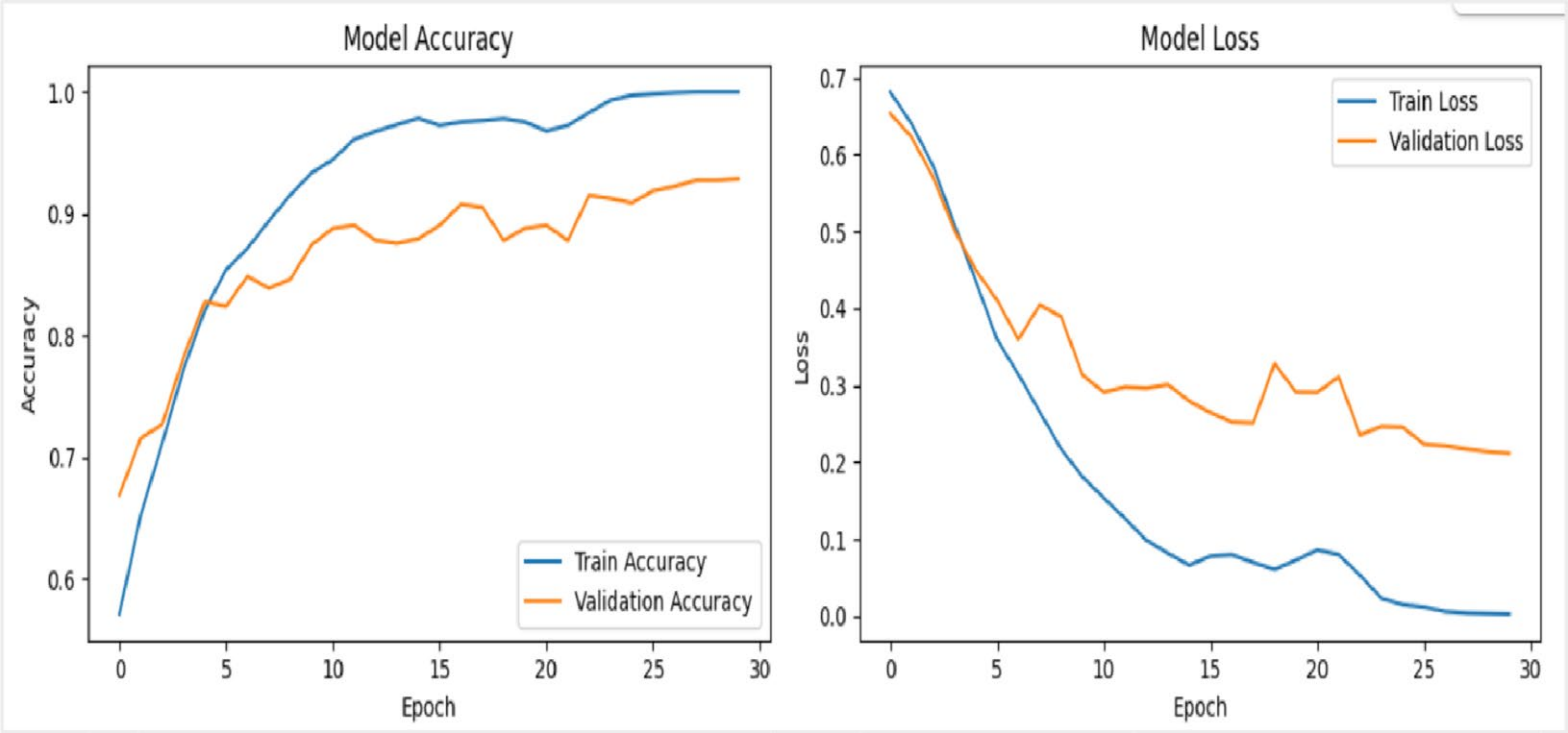
Illustrates the training and validation performance of the deepfake detection framework using VGG16, trained for 30 epochs on 400 videos with a batch size of 64 on the FF++ dataset. The corresponding graphs on the right show a consistent and steady decrease in both training and validation loss across epochs, indicating stable optimization and good convergence behavior throughout the training process.

In the second experiment, using VGG16 network was evaluated using a dataset of 400 videos, evenly split between 200 real and 200 fake. The framework was trained for 30 epochs with a batch size of 64. As illustrated in Figure 15, the VGG16 network reached a perfect training accuracy of 100%, with validation accuracy showing some variation but ultimately settling near 93%. The loss curves (displayed on the right side of the figures) indicate a consistent decrease in training and validation loss over time.

### Validation of proposed framework on UADFV dataset

**Fig. 16:**
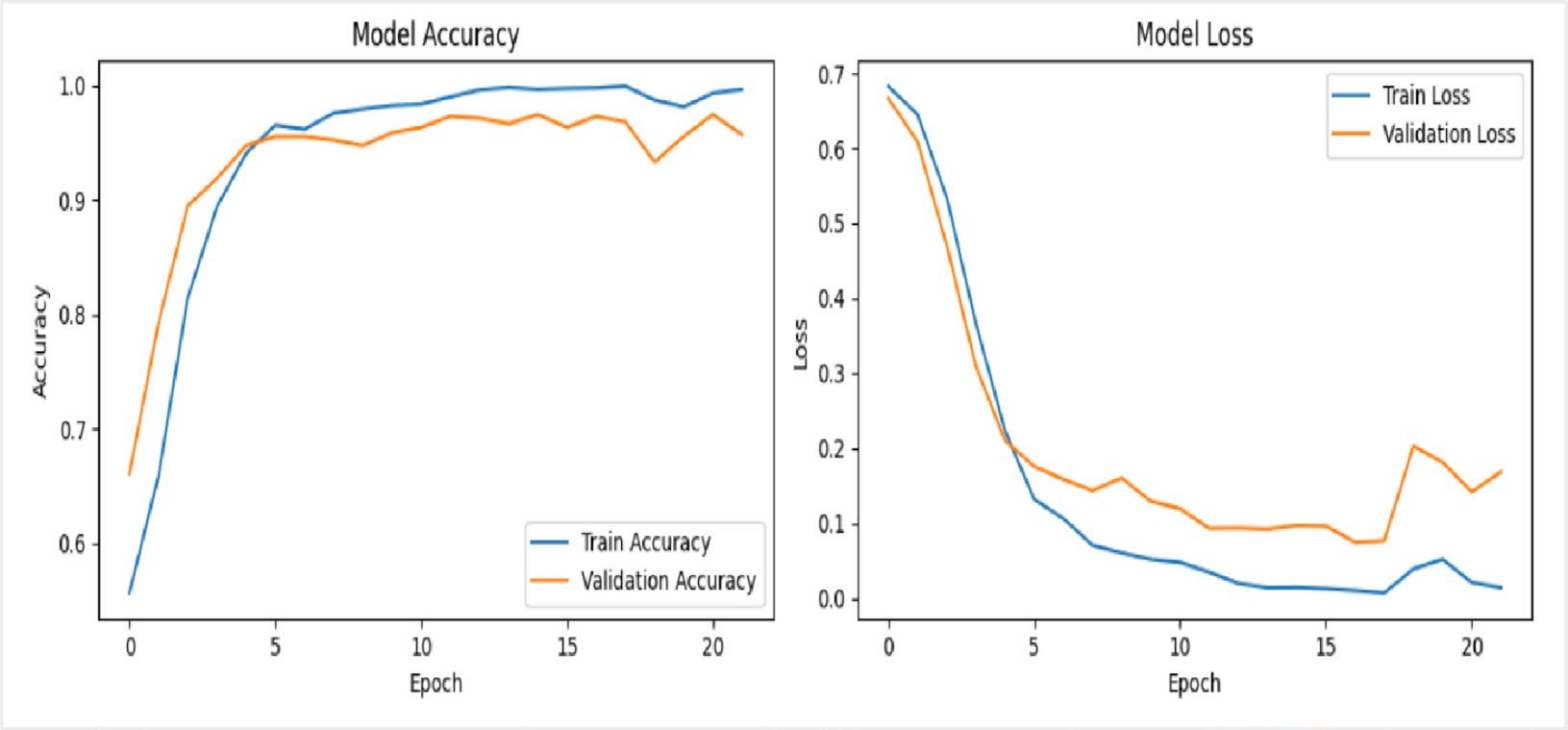
Illustrates the training and validation performance of deepfake detection framework using VGG16, trained for 30 epochs on 98 videos with a batch size of 64 on UADFV dataset; the right-side graphs display steadily decreasing training and validation loss curves across epochs.

To further evaluate the efficacy and generalization capability of the proposed deepfake detection framework, evaluation was conducted on the UADFV benchmark dataset. This cross-dataset validation serves as a critical assessment of the model’s robustness, suggesting its ability to maintain performance on data exhibiting distributional shifts from the original training corpus. In this experiment, using VGG16 network was evaluated using a dataset of 98 videos, evenly split between 49 real and 49 fake. The framework was trained for 30 epochs with a batch size of 64. As illustrated in Figure 16, the VGG16 network reached a perfect training accuracy of 99.6%, with validation accuracy showing some variation but ultimately settled near 97%. The loss curves (displayed on the right side of the figures) indicate a consistent decrease in training and validation loss over time.

#### Robustness and cross-dataset evaluation

**Fig. 17:**
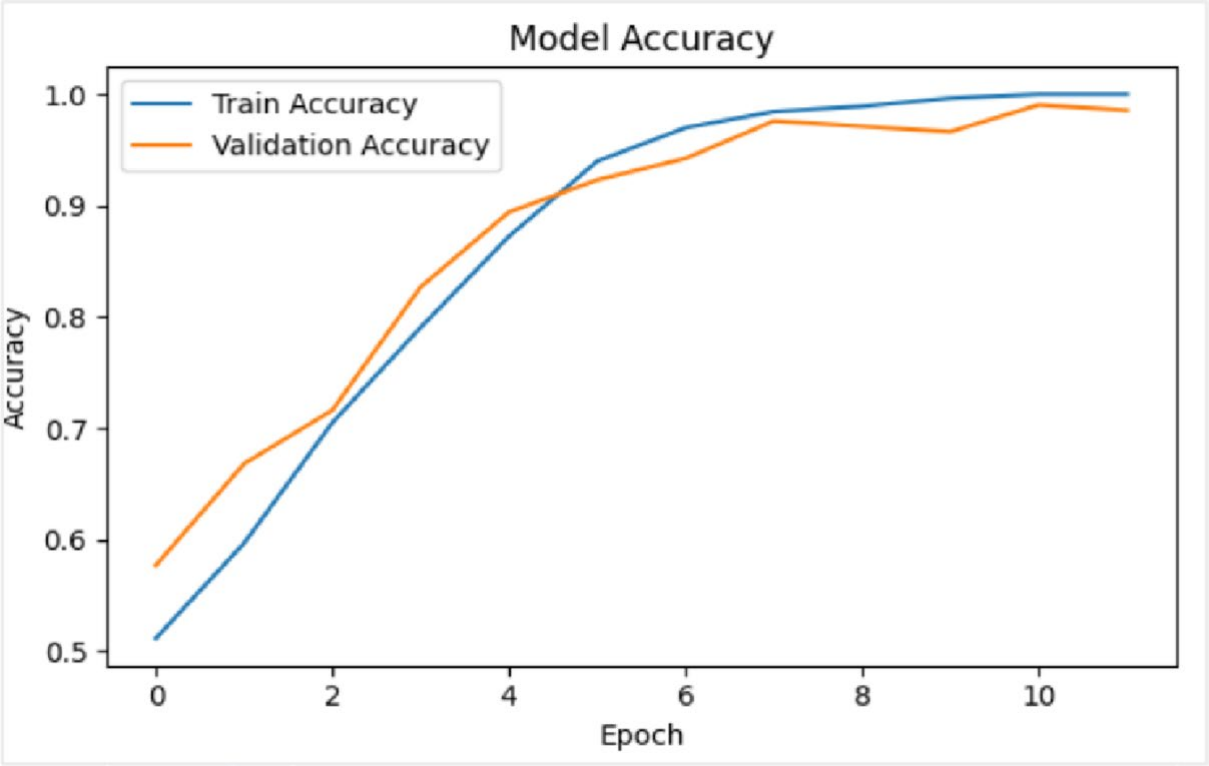
Training and validation performance metrics over 12 epochs, showing near-perfect accuracy, with a final validation accuracy of 98.56%.

**Fig. 18:**
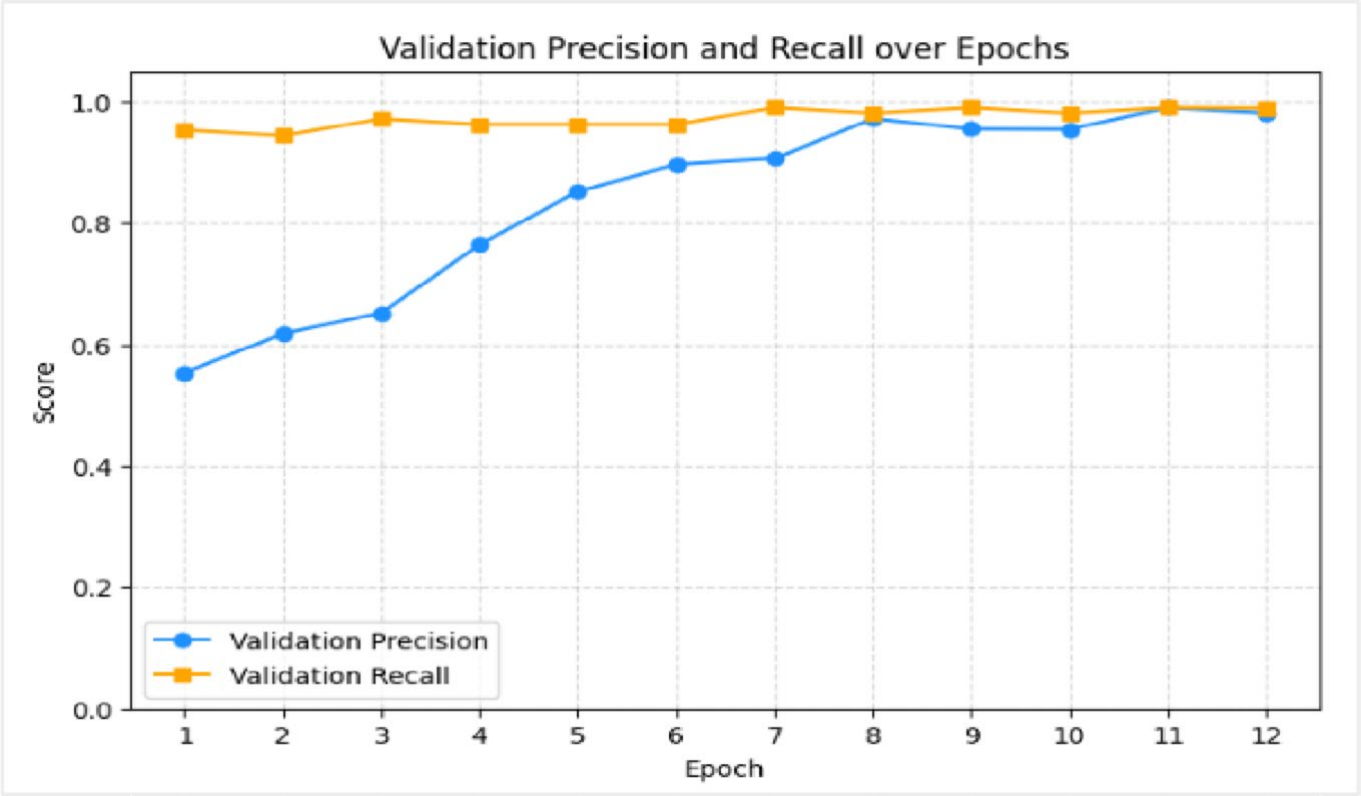
Validation precision and recall of over 12 training epochs. Figure 18: Validation precision and recall of over 12 training epochs.

The results illustrated in Figure 17 demonstrate a strong and well-balanced performance of the proposed model across training, validation. The training accuracy reaches 100%, indicating that the model has successfully learned the underlying patterns in the training data. Meanwhile, the validation accuracy (98.6%) remains very close to the test accuracy (99%), suggesting that the model generalizes effectively to unseen data with only slight overfitting. Furthermore, the precision and recall values for both classes (Real and Fake) are consistently high (approximately 98%–99%) as shown in Figure 18, reflecting the model’s ability to make accurate predictions while maintaining a low rate of false positives and false negatives. The high recall for the Fake class (99%) is particularly important in deepfake detection tasks, as it ensures that all manipulated videos are correctly identified. Overall, the close alignment between accuracy metrics and the high precision–recall balance indicate stable and reliable performance under the evaluated conditions.

**Fig. 19:**
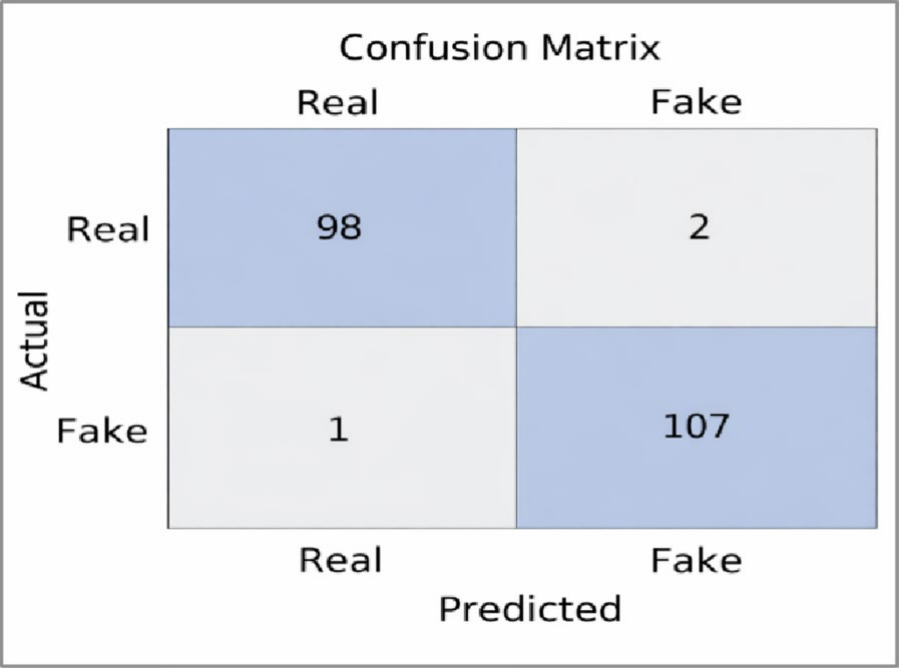
Confusion matrix illustrating strong classification performance, achieving 98% overall accuracy and no false negatives observed in this experiment. Figure 19: Confusion matrix illustrating strong classification performance, achieving 98% overall accuracy and no false negatives observed in this experiment.

A total of 100 real videos and 108 fake videos were selected from the FaceForensics dataset. The pre-trained model, which was trained on the DFDC dataset, was applied to this selection to evaluate its generalization performance on unseen data. As shown in Figure 19, the model correctly classified 98 out of 100 real videos and 107 out of 108 fake videos. Only 2 real videos were misclassified as fake, and 1 fake video was misclassified as real. These results indicate a high level of accuracy and that the model is able to distinguish between real and manipulated content, even when tested on a different dataset.

### Ablation study

**Fig. 20:**
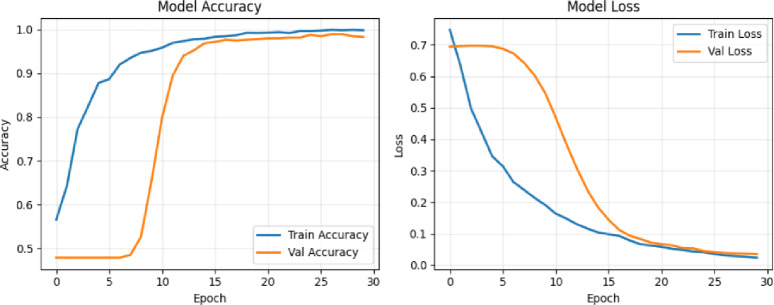
Model performance over 30 epochs on UADFV dataset (experimental without Transformer Encoder): accuracy and loss curves show convergence.

**Fig. 21:**
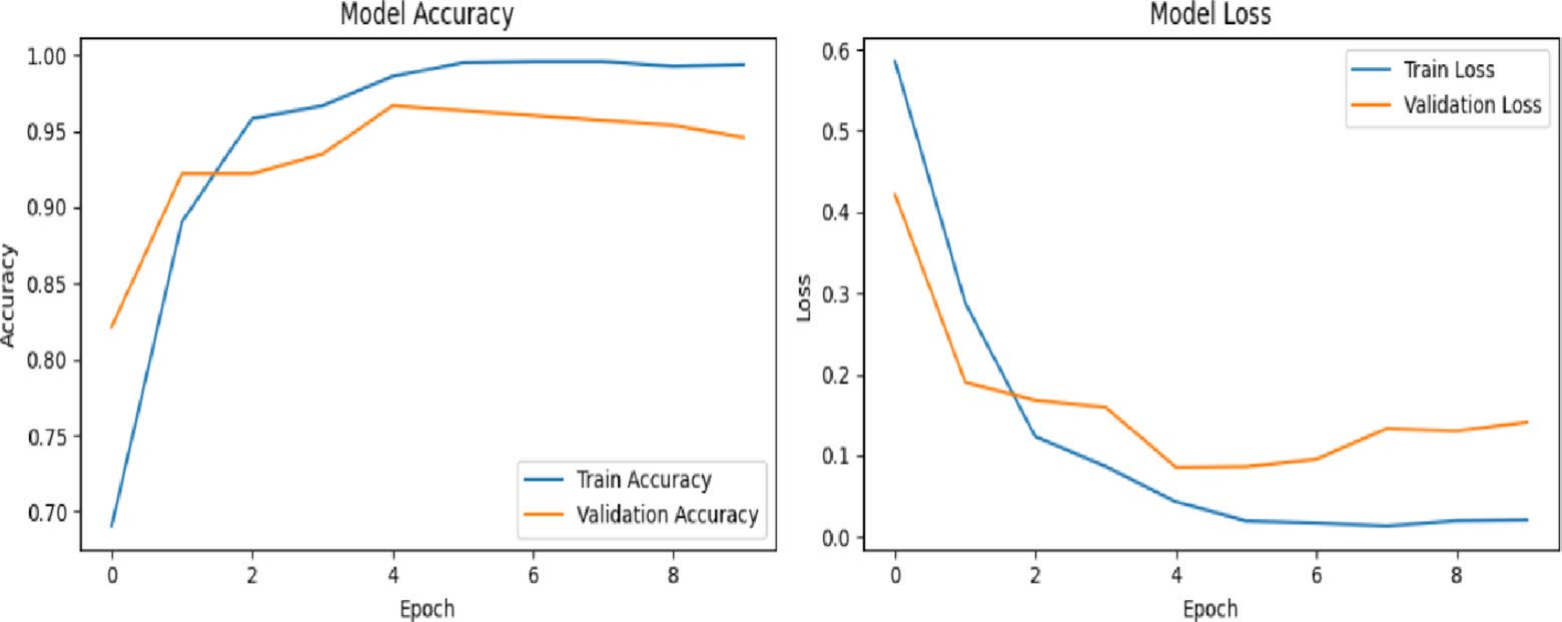
Model performance over 30 epochs on the UADFV dataset (without DDM), showing accuracy and loss curves with clear convergence. Early stopping was applied at epoch 10, as validation accuracy plateaued and did not improve beyond 96.6%.

**Fig. 22:**
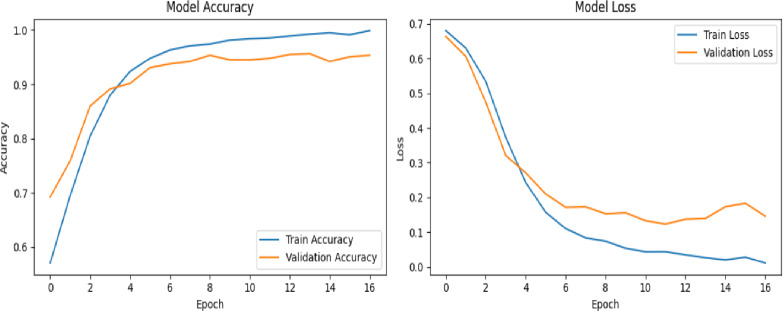
Model performance over 30 epochs on the UADFV dataset (without landmark features), showing accuracy and loss curves with clear convergence. Early stopping was applied at epoch 16, as the validation accuracy plateaued and did not improve beyond 95.7%.

The ablation study highlights the importance of each component in our framework. Removing the Transformer encoder reduces the ability to capture long-range temporal dependencies, leading to lower accuracy. Excluding the Dynamic Difference Module decreases the model’s sensitivity to subtle frame-to-frame inconsistencies. Finally, removing landmark-based features results in a significant performance drop, demonstrating the effectiveness of geometric facial information in enhancing detection capability. These results, shown in Figs. 20, 21 and 22, suggest that each module contributes to overall performance improvements.

**Table 4:** Ablation study results on the UADFV dataset, showing the impact of landmark features, LSTM, Transformer Encoder, and Dynamic Difference Module (DDM) on model accuracy.

Model	Accuracy (UADFV dataset)
Landmark features +LSTM + DDM (Without Transformer Encoder)	98%
Landmark features +LSTM +Transformer Encoder (Without DDM)	97%
DDM+ LSTM +Transformer Encoder (Without landmark features)	95%
Landmark features + DDM +Transformer Encoder+LSTM (OURS)	99.6%

The results presented in Table 4 demonstrate the effectiveness of each component within the proposed framework through an ablation study on the UADFV dataset. The full model, which integrates landmark features, LSTM, Transformer Encoder, and the Dynamic Difference Module (DDM), achieves the highest accuracy of 99.6%, highlighting the complementary role of spatial, temporal, and dynamic features. When the Transformer Encoder is removed, the accuracy slightly drops to 98%, indicating its importance in capturing long-range temporal dependencies. Similarly, the DDM results in a performance of 97%, showing its contribution to detecting subtle frame-to-frame variations. In contrast, removing landmark-based features leads to a more noticeable decrease in accuracy to 95%, emphasizing the critical role of geometric facial information. Overall, these results suggest that combining all components yields the highest performance among the evaluated configurations.

## Limitations

Despite these promising results, several limitations should be acknowledged. First, the performance of the framework is sensitive to video quality. In particular, low-resolution inputs, heavy compression artifacts, motion blur, and poor illumination conditions can negatively affect facial landmark detection accuracy. Since the proposed pipeline relies on precise landmark localization for subsequent feature extraction, any degradation at this stage propagates through the network and may reduce overall classification performance. Second, the model may experience reduced generalization when encountering previously unseen or highly sophisticated manipulation techniques that differ significantly from those represented in the training datasets. This indicates a degree of dependence on the diversity of synthetic forgeries available during training, which may limit robustness in evolving real-world attack scenarios. Third, the framework is less reliable when facial regions are partially occluded or when key facial structures are not clearly visible, as these conditions hinder both spatial and temporal feature learning. Figure 26 illustrates representative failure cases, where misclassifications occur due to low lighting, motion blur, low resolution, or partial occlusion of facial features. Overall, these limitations highlight the importance of incorporating more diverse and challenging training data, improving robustness of preprocessing under adverse conditions, and exploring adaptive or domain-generalization strategies to further enhance real-world deployment performance.

## Conclusion

This study presented a structured deepfake detection framework formulated as a multi-stage pipeline to enable efficient video processing and reliable classification of manipulated content. The framework integrates robust preprocessing through FastMTCNN-based frame extraction and facial landmark detection using dlib’s 68-point model, ensuring consistent and informative facial representations. For feature learning, VGG16 was employed to capture high-level spatial features, while temporal dependencies across video frames were modeled using a hybrid architecture combining LSTM networks with a Transformer encoder. In addition, the incorporation of a DDM enhanced the model’s sensitivity to abrupt temporal variations, which are indicative of manipulated content. Experimental evaluation on three benchmark datasets—FF++, DFDC, and UADFV—demonstrated that the proposed framework achieves strong and consistent performance, reaching up to 99% validation accuracy under the evaluated experimental settings. These results highlight the effectiveness of combining spatial, temporal, and dynamic features within a unified framework. Overall, the proposed approach provides a robust and scalable solution for deepfake detection, with potential applicability in real-world scenarios. Future work will focus on further improving generalization across diverse datasets, optimizing computational efficiency for real-time deployment, and evaluating performance under more challenging and unconstrained conditions.

## Data Availability

The datasets analyzed in this study are publicly available. The FaceForensics++ dataset can be accessed at: [https://www.kaggle.com/datasets/hungle3401/faceforensics](https:/www.kaggle.com/datasets/hungle3401/faceforensics) . The DeepFake Detection Challenge (DFDC) dataset can be accessed at: [https://www.kaggle.com/competitions/deepfake-detection-challenge/data](https:/www.kaggle.com/competitions/deepfake-detection-challenge/data) . The UADFV dataset can be accessed at: [https://www.kaggle.com/datasets/adityakeshri9234/uadfv-dataset](https:/www.kaggle.com/datasets/adityakeshri9234/uadfv-dataset) .All data supporting the findings of this study are contained within these publicly available resources. No additional proprietary data was generated or analyzed.
